# Individualized decision aid for diverse women with lupus nephritis (IDEA-WON): A randomized controlled trial

**DOI:** 10.1371/journal.pmed.1002800

**Published:** 2019-05-08

**Authors:** Jasvinder A. Singh, Liana Fraenkel, Candace Green, Graciela S. Alarcón, Jennifer L. Barton, Kenneth G. Saag, Leslie M. Hanrahan, Sandra C. Raymond, Robert P. Kimberly, Amye L. Leong, Elyse Reyes, Richard L. Street, Maria E. Suarez-Almazor, Guy S. Eakin, Laura Marrow, Charity J. Morgan, Brennda Caro, Jeffrey A. Sloan, Bochra Jandali, Salvador R. Garcia, Jennifer Grossman, Kevin L. Winthrop, Laura Trupin, Maria Dall’Era, Alexa Meara, Tara Rizvi, W. Winn Chatham, Jinoos Yazdany

**Affiliations:** 1 University of Alabama at Birmingham, Birmingham, Alabama, United States of America; 2 Birmingham VA Medical Center, Birmingham, Alabama, United States of America; 3 Yale University, New Haven, Connecticut, United States of America; 4 Oregon Health Science University, Portland, Oregon, United States of America; 5 VA Portland Health Care System, Portland, Oregon, United States of America; 6 Lupus Foundation of America, Washington, DC, United States of America; 7 Healthy Motivation, Inc., Los Angeles, California, United States of America; 8 Elyse Reyes Consulting, Los Angeles, California, United States of America; 9 Texas A&M University, College Station, Texas, United States of America; 10 University of Texas MD Anderson Cancer Center, Houston, Texas, United States of America; 11 Arthritis Foundation, Atlanta, Georgia, United States of America; 12 Georgia State University, Atlanta, Georgia, United States of America; 13 Mayo Clinic School of Medicine, Rochester, Minnesota, United States of America; 14 Baylor College of Medicine, Houston, Texas, United States of America; 15 University of California, Los Angeles (UCLA), Los Angeles, California, United States of America; 16 University of California at San Francisco (UCSF), San Francisco, California, United States of America; 17 Ohio State University, Columbus, Ohio, United States of America; Royal Derby Hospital, UNITED KINGDOM

## Abstract

**Background:**

Treatment decision-making regarding immunosuppressive therapy is challenging for individuals with lupus. We assessed the effectiveness of a decision aid for immunosuppressive therapy in lupus nephritis.

**Methods and findings:**

In a United States multicenter, open-label, randomized controlled trial (RCT), adult women with lupus nephritis, mostly from racial/ethnic minority backgrounds with low socioeconomic status (SES), seen in in- or outpatient settings, were randomized to an individualized, culturally tailored, computerized decision aid versus American College of Rheumatology (ACR) lupus pamphlet (1:1 ratio), using computer-generated randomization. We hypothesized that the co-primary outcomes of decisional conflict and informed choice regarding immunosuppressive medications would improve more in the decision aid group. Of 301 randomized women, 298 were analyzed; 47% were African-American, 26% Hispanic, and 15% white. Mean age (standard deviation [SD]) was 37 (12) years, 57% had annual income of <$40,000, and 36% had a high school education or less. Compared with the provision of the ACR lupus pamphlet (*n* = 147), participants randomized to the decision aid (*n* = 151) had (1) a clinically meaningful and statistically significant reduction in decisional conflict, 21.8 (standard error [SE], 2.5) versus 12.7 (SE, 2.0; *p* = 0.005) and (2) no difference in informed choice in the main analysis, 41% versus 31% (*p* = 0.08), but clinically meaningful and statistically significant difference in sensitivity analysis (net values for immunosuppressives positive [in favor] versus negative [against]), 50% versus 35% (*p* = 0.006). Unresolved decisional conflict was lower in the decision aid versus pamphlet groups, 22% versus 44% (*p* < 0.001). Significantly more patients in the decision aid versus pamphlet group rated information to be excellent for understanding lupus nephritis (49% versus 33%), risk factors (43% versus 27%), medication options (50% versus 33%; *p* ≤ 0.003 for all); and the ease of use of materials was higher in the decision aid versus pamphlet groups (51% versus 38%; *p* = 0.006). Key study limitations were the exclusion of men, short follow-up, and the lack of clinical outcomes, including medication adherence.

**Conclusions:**

An individualized decision aid was more effective than usual care in reducing decisional conflict for choice of immunosuppressive medications in women with lupus nephritis.

**Trial registration:**

Clinicaltrials.gov, NCT02319525.

## Abstract 2

Jasvinder Singh and colleagues reveal how a decision aid helps women with lupus to make decisions about immunosuppressive therapy.

## Author summary

**Why was this study done?:**

- Lupus kidney disease is a serious immune system condition that can lead to kidney failure and dialysis in young women, if not treated appropriately. Effective treatment with immune-blocking medicines is available, but patients report difficulty making informed decisions that weigh benefits and risks, such as side effects.
- No patient decision aids are available for lupus medication decision-making.
- The research team made an online decision aid for women with lupus kidney disease, with extensive input from individuals with lupus. The aid can be individualized to help patients make informed decisions about immune-blocking medicines for lupus.

**What did the researchers do and find?:**

- In 301 women with lupus kidney disease across four centers, researchers assessed whether an online decision aid improved lupus treatment decisions, compared with the use of a paper pamphlet on lupus kidney disease from the American College of Rheumatology.
- Compared with women who received the lupus pamphlet, women who used the decision aid reported feeling less doubt about their medication choice.
- More women who used the online decision aid said that the information source was easy to use, compared with women who used the lupus pamphlet.

**What do these findings mean?:**

- This study provides evidence that a decision aid for women with kidney disease may help patients feel more confident in their treatment choices.

## Introduction

Systemic lupus erythematosus (SLE) is a chronic disease primarily affecting young women, with significant morbidity and mortality. Compared with whites, African-Americans and Hispanic groups have higher SLE incidence, worse disease severity and outcomes [1,2], and greater mortality [3]. Approximately 35% of SLE patients present with lupus nephritis and 50%–60% develop it within 10 years [4,5]. Lupus nephritis accounts for 2% of end-stage renal disease (ESRD) in the United States [6]. It is significantly more prevalent and has worse outcomes in African-Americans and Hispanic groups [4,7].

Treatment of lupus nephritis with immunosuppressive medications is complex, especially for young women, and carries risks of infertility, teratogenicity, and serious infections. Many patients face difficult decisions, necessitating clear patient–provider communication and shared decision-making. Decision aids can support shared decision-making to ensure that treatment plans are consistent with patients’ values [8]. Moreover, patient participation in decision-making can improve outcomes [9,10], including medication adherence [11–14]. Adherence to lupus medications, including immunosuppressive drugs, is lower in women of racial/ethnic groups [15,16]. Low adherence is associated with poorer outcomes [17]. These data suggest that a decision aid may increase patient participation in decision-making and ultimately improve adherence in vulnerable patient populations, who are less engaged in their care.

To our knowledge, no lupus decision aids are available. In the U.S., 41% of Hispanic groups, 24% of African-Americans, and 9% of whites have below basic health literacy skills [18]. Lower health literacy and numeracy are associated with greater risk aversion [19] and may interfere with the delivery of guideline-concordant care in racial/ethnic minorities with lupus. As an example, many patients decline immunosuppressive medications due to fear of side effects and lack of recognition of benefits, including prevention of ESRD [20]. Most lupus educational materials are written at readability levels above the recommended sixth grade reading level and have only adequate suitability (no assessment of numeracy level) [21]. Decision aids that address patients’ literacy and numeracy levels are therefore warranted.

Based on qualitative work with patients [22–24] and the comparative effectiveness research (CER) data on benefits and risks of immunosuppressive medications in lupus nephritis [25–27], we developed an individualized, culturally tailored, computerized decision aid for medication decision-making for patients with lupus nephritis. The lupus decision aid was created in the first year followed by its testing in years 2–3 of a 3-year Patient-Centered Outcomes Research Institute (PCORI) contract; the details of decision aid development are available in a previous publication [28].

We assessed whether patients randomized to an individualized, culturally and linguistically appropriate, computerized decision aid were more likely to make more informed treatment decisions compared with patients randomized to an American College of Rheumatology (ACR) patient information lupus pamphlet. This randomized controlled trial (RCT), the Individualized Decision aid for Diverse Women with Lupus Nephritis (IDEA-WON) study, tested the efficacy of our individualized lupus decision aid. We hypothesized that the lupus decision aid would lead to (1) a greater reduction in decisional conflict and a higher likelihood of making an informed choice (co-primary outcomes); (2) less discordance in patients’ preferred role in decision-making between the desired versus actual role in decision-making, and improved patient–physician communication (secondary outcomes); and (3) being acceptable and feasible. The study was designed to capture populations most affected by lupus nephritis by involving centers that serve large numbers of vulnerable populations. Our study included predominantly African-American and Hispanic subjects, because lupus nephritis is more prevalent and more severe in women from minority backgrounds [4,7].

## Methods

### Study population, study sites, randomization, and Clinicaltrials.gov registration

We conducted a multicenter, parallel, two-arm, open-label RCT, comparing the ACR lupus paper pamphlet (**S1 Text**) to an individualized, culturally and linguistically appropriate decision aid for women with lupus nephritis called shared decision-making in lupus electronic tool (SMILE). All outcomes were patient assessed and patient reported, and neither patients nor assessors were blinded. Women with lupus nephritis were recruited from four geographically diverse sites (University of Alabama at Birmingham [UAB], University of California at San Francisco [UCSF], Baylor College of Medicine, and Ohio State University). After obtaining written informed consent, we randomized participants using a computer-generated randomization process based upon a permuted variable block design, stratified by study site and language (English versus Spanish), and designed by a biostatistician. Our study was registered at Clinicaltrials.gov (NCT02319525) and was approved by each of the participating sites’ Human Subjects Studies Programs. Our trial protocol published elsewhere provides additional details of the study protocol and the development of the decision aid with extensive multi-stakeholder input, including patients, clinicians, patient advocacy organization leaders, and researchers [28]. This study is reported as per the Consolidated Standards of Reporting Trials (CONSORT) guideline (S1 CONSORT Checklist).

### Subject eligibility, recruitment, and retention

Adult women (≥18 years) of all race/ethnicities currently having a lupus nephritis flare and considering change or initiation of an immunosuppressive medication (current flare) or who had a prior lupus nephritis flare and were at risk for a future lupus nephritis flare (at risk for nephritis flare) were eligible. Lupus nephritis flare was defined as an increase in disease activity, indicated by an increase in proteinuria and/or serum creatinine concentration, abnormal urine sediment, or a reduction in creatinine clearance rate as a result of active disease, similar to previous studies [29,30,31,32] and as defined by the ACR lupus nephritis guideline [33]. A lupus nephritis flare is an indication for initiation or change of immunosuppressive medication. Study exclusions were male sex, dialysis, renal transplant, or planned renal transplant (**S2 Text**). Initial enrollment of only African-American and Hispanic women (given our focus on racial/ethnic minorities) was expanded to also include white and Asian women to increase the generalizability of the study findings and increase enrollment.

Participants were identified through screening outpatient clinic lists for diagnostic codes for lupus nephritis in the clinical electronic health record (EHR) databases or by direct physician referral of people with a new diagnosis of lupus nephritis in in- or outpatient setting. Research associates then conducted a medical record review to confirm eligibility using the preceding inclusion/exclusion criteria; all patients met the 1997 ACR revised classification criteria for lupus [34] and were diagnosed with lupus nephritis by a rheumatologist based on the presence of proteinuria, urinary casts, a kidney biopsy, and/or other laboratory tests (creatinine, blood urea nitrogen, etc.). The research associates obtained written informed consent and Health Insurance Portability and Accountability Act (HIPAA) authorization from each participant prior to study participation and conducted study visits during the patient’s regularly scheduled outpatient visits. Participants were enrolled from March 15, 2015, to November 4, 2016. All patients were recruited at routine outpatient clinic appointments, and all study procedures were completed at routine clinic appointments.

### Intervention: Individualized, culturally appropriate, computerized decision aid versus pamphlet

Patients with lupus nephritis were randomized in a 1:1 ratio to the provision of the decision aid or the ACR lupus paper pamphlet (**S1 Text**) in the doctor’s office after completing baseline pre-intervention assessments (demographics, health literacy, numeracy, etc.). Follow-up assessments were kept to a minimum due to the nature of the study and to minimize missing data. At 3 months, study subjects assessed patient–physician communication either during a routine clinic visit, via phone (if no clinic visit), or via mail (if not reachable via phone and in clinic).

The decision aid was developed based on the International Patient Decision aid Standards (IPDAS) [35] with multi-stakeholder group input (individuals with lupus, patient coinvestigators, clinicians, and researchers) and underwent iterative modification and pilot testing. It was tailored to the target population’s numeracy and health and graphical literacy levels [36]. It incorporated barriers to and facilitators of medication decision-making in women with lupus nephritis [22–24] and the CER data on medication benefits and risks [25–27]. Themes generated from nominal groups of patients with lupus nephritis, including African-American, Hispanic, Asian, and white women [22–24], were incorporated into the decision aid content and presentation. Because we recruited similar target patient populations for the nominal groups and the trial (those from racial/ethnic minority groups with low socioeconomic status [SES] or low literacy), themes and content generated were culturally tailored to included populations. Additionally, patient research partners and patient advocacy leaders (study coinvestigators) consisted of racial/ethnic minorities as well as white women, who reviewed the lupus decision aid content for cultural appropriateness and provided feedback.

Individualization of the decision aid (**S1 Fig**) was done in several ways. We provided specific immunosuppressive medication comparisons based on the options being considered (or possible to be considered in the future), given the treatment phase (induction versus maintenance) and the current treatment(s) (**S1 Fig**) [28]. We gave optional links to additional information embedded in the decision aid, including sections on pregnancy, breastfeeding, fertility, and glucocorticoids side effects (**S1 Fig**). Optional links on how to manage specific adverse events associated with immunosuppressive medications were provided. We also provided the decision aid in both English and Spanish to allow patients to view it in the language they choose. Images of the lupus decision aid are provided as supporting information (**S1 Fig**). The decision aid was administered using tablet computers.

All patients randomized to the decision aid read information describing disease manifestations, with relative benefits and harms for different treatments options based on their personal histories. The decision aid also included links to support groups. Participants were able to stop, rewind, and review the content. The co-primary and secondary outcomes were measured after administration of the decision aid/pamphlet, followed by the clinic visit with the healthcare provider, which was audiotaped (**S3 Text**). The informed consent, pamphlet, decision aid, and all data collection materials were available in English and Spanish. The control group received the ACR patient information lupus pamphlet (**S1 Text**) that provided information about lupus and its treatment, including the use of immunosuppressive drugs.

### Study outcomes

Co-primary outcomes were change in the decisional conflict score and the proportion with an informed choice post-intervention; secondary outcomes were physician–patient communication measures and patient preference for decision-making (see **S3 Text**). Decisional conflict was measured using the low literacy version of the Decisional Conflict Scale (DCS), a well-validated self-administered instrument [37]. The low literacy version has 10 items with 3 response categories: yes, unsure, and no. Four subscale scores consisting of uncertainty about choice, feeling informed, values clarity, and feeling supported in decision-making were also calculated. DCS (and subscale) scores range from 0 (best) to 100 (worst) and scores ≥25 are consistent with clinically significant residual decisional conflict [38].

Informed value-concordant choice was assessed using a validated multidimensional model of informed choice [39,40] that individually assessed and then combined three constructs regarding immunosuppressive medications: values (favoring or against) [41], objective knowledge [42], and choice (decision to or not to start immunosuppressive medication) [43]. Value (10 value statements; for detailed methods, see **S4 Text**) and knowledge (20 true/false questions; **S4 Text**) items were developed based on the results of a previously conducted nominal group study [23,24]. Participants were classified into those favoring versus against the use of immunosuppressive medications using the median value score (negative values were reverse coded). Choice predisposition towards starting immunosuppressive medications was assessed using a 15-point scale (anchor scores were 1 [willing] and 15 [not willing] and uncertain in the center) in response to, “If your doctor recommended that you take an immunosuppressive drug for your lupus nephritis, would you be willing to take one?” Participants’ choices were classified as “willing” (1–7), “undecided” (8), or “unwilling” (9–15). Informed choice refers to one based on adequate knowledge (score of ≥75%) and concordant with one’s values related to immunosuppressive medications (favoring or against).

In contrast to the main analysis, in which we categorized values above or below the median as favoring/not favoring immunosuppressive medications (statistical approach), we performed a sensitivity analysis for informed choice by reclassifying participants according to the net score as positive or negative on value statements (clinical approach), comparing value statements favorable towards immunosuppressive medications (e.g., “Taking medicine now is important to increase my chance of being healthy in the future”—positive values) with value statements that were not favorable (e.g., “It is not a good idea to take medicines for years”—negative values). This was an a priori protocol-specified analysis [28].

Preferred role in decision making was assessed using the Control Preferences Scale [44]. We used this instrument to ask participants their preferred role as well as the actual role they played. The latter was asked only in those with a current lupus nephritis flare. We classified responses into active (active/active shared), collaborative, or passive roles (passive/passive shared) [44].

Patient–physician communication and care processes were assessed using the Interpersonal Processes of Care short form (IPC-SF), an 18-item validated patient-reported patient–physician communication and care processes measure [45]. Scores range from 18 to 90, with higher scores indicating better communication/care.

Analysis of the audiotaped baseline physician–patient visit was performed using the Active Patient Participation Coding Scheme (APPC), a validated instrument to assess indicators and facilitators of patient participation [46]. Clinic visits were only audiotaped and coded among participants with a current flare and who agreed to be recorded. We assessed active patient participation and patient-centered communication by the doctor[47]. Each utterance was coded by trained coders and scored, with higher scores indicating better patient participation/communication.

Acceptability of the decision aid/pamphlet (information quality and quantity, presentation style, and usefulness) was assessed using a validated acceptability survey [48] on a 4-point scale ranging from “excellent” to “poor.” Feasibility of the decision aid/pamphlet and the study procedures was assessed with a self-administered questionnaire [49]. Participants rated the ease of using the decision aid/pamphlet, survey comprehension, content and readability, and the time needed to review the decision aid/pamphlet and questionnaires on 5-point agreement scales.

### Study covariates

In addition to race/ethnicity, education, income, and language, the following were assessed: (1) health literacy, using the validated 18-item Short Assessment of Health Literacy tool (SAHL-E and SAHL-S) [50], for which the number of words read and associated correctly are summed (possible range = 0–18; low health literacy = 0–14); (2) subjective numeracy, using an 8-question self-administered scale [51] (possible range = 1–6; low subjective numeracy = 0–3); (3) graphical literacy, using the short form version by Galesic and colleagues [52] (possible range = 0–4; low graphical literacy = 0–3); and (4) trust in physicians using the validated 11-item self-administered scale [53,54], in which responses are coded on a 5-point Likert scale (totally disagree to totally agree; possible range = 11–55; low trust = 11–43).

### Statistical analyses

Statistical analyses were performed using SAS software (SAS version 9.4, Cary, NC). We used two-sample *t* tests to compare study arms for continuous outcomes (change in DCS scores; secondary outcomes, IPC-SF score, and audiotaped physician–patient interaction scores), and chi-squared test for categorical outcomes (informed choice; secondary outcome, role concordance on control preferences scale; acceptability; and feasibility). To control for possible baseline imbalances in decisional conflict and informed choice, we also used analysis of covariance (ANCOVA) and logistic regression to compare post-intervention decisional conflict and informed choice, respectively, accounting for baseline values (pre-intervention decisional conflict and pre-intervention knowledge, values, and choice, respectively).

We examined treatment heterogeneity by performing subgroup analyses by language, race/ethnicity, SES, type of scenario (current flare versus at risk for flare), numeric literacy, income, health literacy, graphical literacy, and trust in physicians score, using linear (decisional conflict) and logistic (informed consent) regression models, adjusting for baseline covariates. As some of these analyses were not prespecified (graphical literacy, trust in physicians score), a Bonferroni correction was used to account for multiple testing for all analyses, as a conservative approach.

The primary analysis was on an intent-to-treat basis. All *p*-values were two sided, and *p* < 0.05 was considered statistically significant, except subgroup analyses in which we used the Bonferroni correction to account for multiple testing (*p* < 0.0008 considered significant [= 0.05/63]). We considered a 10% difference between study arms in the proportion of patients achieving a favorable or unfavorable outcome to be clinically meaningful. We assessed whether change in knowledge versus clarification of choices mediated the change in decisional conflict related to decision aid, assessed using the mediation analyses [55].

Our proposed sample size of 200 patients, with 90 patients in each study arm (10% loss to follow-up; 45 each Hispanic and African-American), had an 80% power to detect a large effect size difference of 0.60 between group means on the DCS score using a two-sided type I error rate of 0.05 and a 21% difference in informed choice using a one-sided type I error rate of 0.05, based on published results, 12% in the usual care versus 33% in the decision aid group [56]. An effect size of 0.4–0.8 represents a clinically meaningful difference in the DCS, discriminating between those who make and delay decisions [57]. Because of a low recruitment rate of Hispanic women, we enrolled 301 participants, aiming to have as close to 90 Hispanic patients for analyses as possible. The study protocol was modified to recruit white and Asian participants to improve generalizability.

### Patient and public involvement

Two patients and four patient advocacy organization leaders (LMH, SCR, GSE, LM) were study coinvestigators who participated as key stakeholders in study design and conduct, reviewed results, and coauthored the study. Qualitative work with patients to define the content and focus of the decision aid [22–24], and extensive piloting of decision aid [28], helped us maintain a strong patient-centered focus.

### Ethical approval and consent to participate

The UAB’s Institutional Review Board approved this study, and all investigations were conducted in conformity with ethical principles of research. The study was also approved by the Institutional Review Boards at each of the other study sites, including the Baylor College of Medicine, Houston, TX, the Ohio State University, Columbus, OH, and UCSF, San Francisco, CA.

## Results

### Baseline patient characteristics

**Fig 1:**
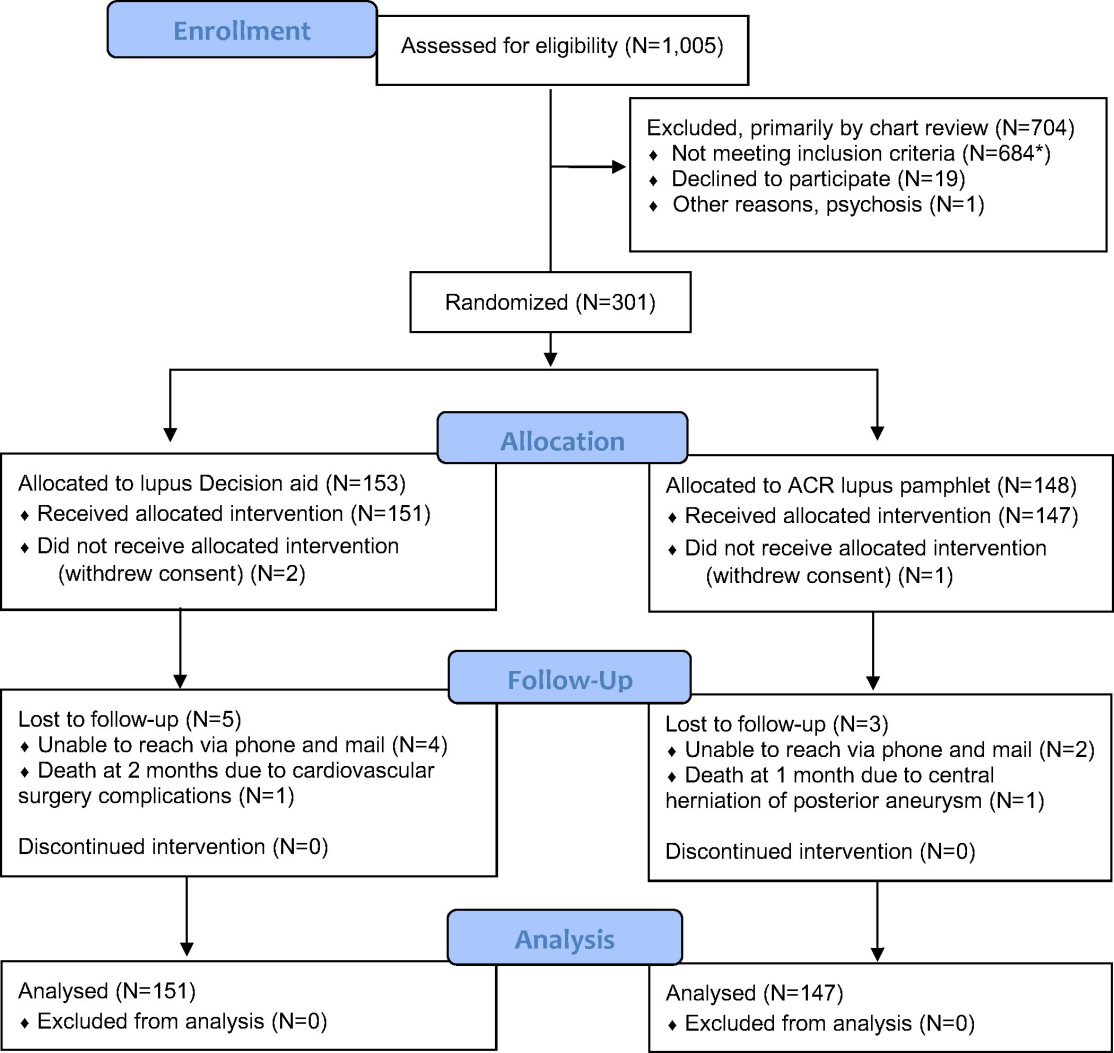
CONSORT diagram for patient selection. The figure includes 1,005 patients prescreened using electronic medical records and/or in-person screening. Of these, 704 were excluded, 19 declined to participate (12 had no time; 5 were not interested in participating in a research study, and 2 did not want any more information about medications for lupus), and 1 was excluded due to psychosis; An asterisk (*) indicates that the remaining 684 did not meet eligibility, and the reasons were as follows: no nephritis (*n* = 388); candidate for or already had renal transplant (*n* = 162); dialysis (*n* = 99); potential but no clinic appointments (*n* = 29); and miscellaneous (*n* = 6): needed biopsy (*n* = 1); kidney disease not due to lupus (*n* = 1); lupus complications, nonrenal (*n* = 1); none of the scenario matches any treatment option for the patient (*n* = 1); and not sufficient evidence from clinic notes (*n* = 2). ACR, American College of Rheumatology.

Of the 301 participants enrolled in 2015–2016, three withdrew consent before receiving either study intervention; 298 participants randomized to the decision aid (*n* = 151) or pamphlet (*n* = 147) generated data (**Fig 1, CONSORT** diagram). The mean age (standard deviation [SD]) was 37 (SD, 12) years, 57% had annual incomes of less than $40,000, 36% had high school educations or less, 34% were married, and 85% were nonwhite. The average health literacy score was 16.8 (SD, 2.5). Characteristics by study arm are described in **Table 1**. There were no significant baseline differences between the groups, except the difference in immunosuppressive choice (**Table 1**).

**Table 1:** Baseline demographic and clinical characteristics of study participants.

Characteristics	All (*n* = 298) Mean ± SEM or *N* (%)	Decision Aid (*n* = 151) Mean ± SEM or *N* (%)	Pamphlet (*n* = 147) Mean ± SEM or *N* (%)	*p*- value
Age in years	37.3 ± 0.7	37.1 ± 1.0	37.6 ± 1.0	0.72
Race/Ethnicity, *n* (%)				0.73
Non-Hispanic Black	141 (47.3%)	70 (46.4%)	71 (48.3%)	
Hispanic/Latino	78 (26.2%)	41 (27.1%)	37 (25.2%)	
Non-Hispanic White	44 (14.8%)	20 (13.2%)	24 (16.3%)	
Asian	20 (6.7%)	11 (7.3%)	9 (6.1%)	
Other	13 (4.4%)	7 (4.6%)	6 (4.1%)	
Not answered	2 (0.7%)	2 (1.3%)	—	
Marital status				0.39
Don't know/Not answered	3 (1.0%)	3 (2.0%)	—	
Widowed	5 (1.7%)	3 (2.0%)	2 (1.4%)	
Never married	145 (48.7%)	76 (50.3%)	69 (46.9%)	
Divorced or separated	43 (14.4%)	19 (12.6%)	24 (16.3%)	
Married or living with a partner	102 (34.2%)	50 (33.1%)	52 (35.4%)	
Education				0.10
Don't know/Not answered	3 (1.0%)	3 (2.0%)	—	
High school or less	106 (35.6%)	48 (31.8%)	58 (39.5%)	
Greater than high school	189 (63.4%)	100 (66.2%)	89 (60.5%)	
Employment				0.25
Retired	8 (2.7%)	5 (3.3%)	3 (2.0%)	
Working	111 (37.2%)	54 (35.8%)	57 (38.8%)	
Keeping house	35 (11.7%)	17 (11.3%)	18 (12.2%)	
Unable to work	91 (30.5%)	45 (29.8%)	46 (31.3%)	
Going to school	15 (5.0%)	4 (2.6%)	11 (7.5%)	
Looking for work	12 (4.0%)	8 (5.3%)	4 (2.7%)	
Had a job, but not working	9 (3.0%)	5 (3.3%)	4 (2.7%)	
Other	16 (5.4%)	12 (8.0%)	4 (2.7%)	
Don't know/Not answered	1 (0.3%)	1 (0.7%)	—	
Annual household Income				0.57
Less than $40,000	169 (56.7%)	89 (58.9%)	80 (54.4%)	
$40,000–$80,000	44 (14.8%)	18 (11.9%)	26 (17.7%)	
$80,000 or more	32 (10.7%)	17 (11.3%)	15 (10.2%)	
Don't know/Not answered	53 (17.8%)	27 (17.9%)	26 (17.7%)	
Size of household	3.21 ± 0.1	3.4 ± 0.2	3.02 ± 0.1	0.12
Language (survey, decision aid)				0.69
English	255 (85.6%)	128 (84.8%)	127 (86.4%)	
Spanish	43 (14.4%)	23 (15.2%)	20 (13.6%)	
Flare type				0.88
Current	68 (22.8%)	35 (23.2%)	33 (22.4%)	
Future	230 (77.2%)	116 (76.8%)	114 (77.5%)	
Health literacy—SAHL	16.9 ± 0.1	16.8 ± 0.2	16.88 ± 0.2	0.87
Subjective Numeracy Scale	3.8 ± 0.1	4.0 ± 0.1	3.75 ± 0.1	0.11
Ability Subscale	3.8 ± 0.1	3.9 ± 0.1	3.64 ± 0.1	0.11
Preference Subscale	3.9 ± 0.1	4.0 ± 0.1	3.86 ± 0.1	0.24
Short Graph Literacy Scale	1.6 ± 0.1	1.6 ± 0.1	1.63 ± 0.1	0.99
Trust in Physician Scale	46.5 ± 0.4	46.2 ± 0.5	46.81 ± 0.5	0.42
**Pre-intervention DCS score**	35.4 ± 1.7	33.4 ± 2.4	37.48 ± 2.5	0.23
Uncertainty subscale	35.0 ± 2.2	33.5 ± 3.2	36.56 ± 3.2	0.49
Informed subscale	44.9 ± 2.2	42 ± 3.1	47.96 ± 3.2	0.18
Values clarity subscale	39.7 ± 2.3	37.8 ± 3.3	41.67 ± 3.4	0.41
Support subscale	23.2 ± 1.7	21.7 ± 2.5	24.83 ± 2.3	0.36
Pre-intervention unresolved clinically significant decisional conflict on DCS (score ≥25)	178 (59.7%)	85 (56.3%)	93 (63.3%)	0.31
**Pre-intervention informed choice for immunosuppressives**				0.12
Informed	84 (28.2%)	49 (32.4%)	35 (23.8%)	
Not informed	213 (71.5%)	101 (66.9%)	112 (76.2%)	
**Pre-intervention informed choice components**				
Knowledge (percentage correct)	74.6 ± 0.7	74.3 ± 0.9	74.8 ± 1.0	0.72
Values for immunosuppressives				0.98
Against	134 (45.0%)	68 (45.0%)	66 (44.9%)	
In favor	164 (55.0%)	83 (55.0%)	81 (55.1%)	
Choice for immunosuppressives				0.01
Undecided	115 (38.6%)	47 (31.3%)	68 (46.3%)	
Unwilling	34 (11.4%)	23 (15.3%)	11 (7.5%)	
Willing	148 (49.7%)	80 (53.3%)	68 (46.3%)	

Missing: SAHL was missing for 3 decision aid, 1 pamphlet; Short Graph Literacy was missing for 2 decision aid, 1 pamphlet; Pre-intervention informed choice missing for 1 decision aid; Choice for immunosuppressives at baseline was missing for 1 decision aid.

Abbreviations: DCS, Decisional Conflict Scale; SAHL, Short Assessment of Health Literacy.

### Primary outcomes

**Fig 2:**
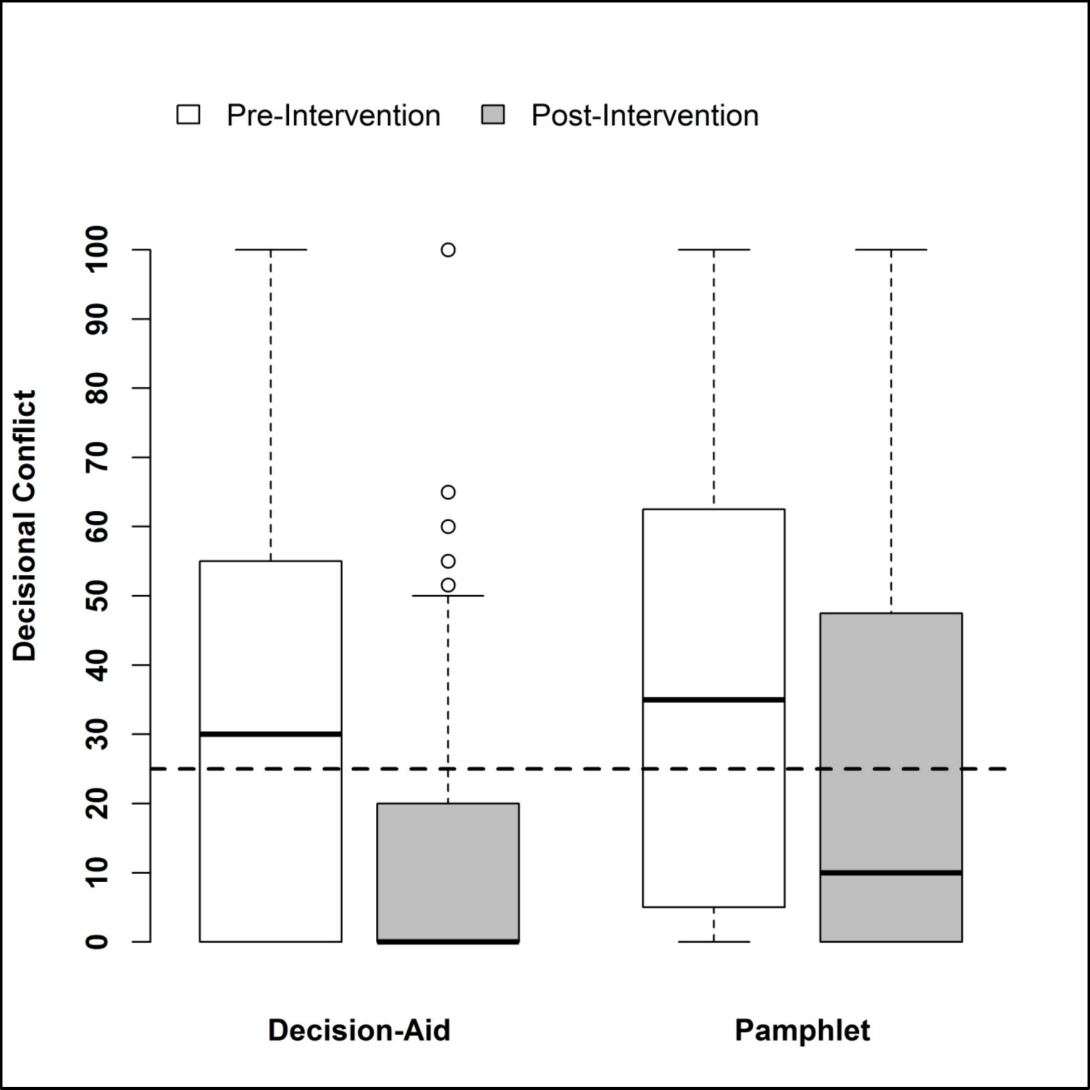
Pre and Post-intervention decisional conflict (0–100; higher score indicates more conflict). Dashed line represents the threshold for unresolved clinically significant decisional conflict (≥25) on the DCS. The box plot shows the median, indicated by the solid line, and 25th and 75th percentiles as the lower and upper bounds of the box. The whiskers represent the minimum and maximum values. DCS, Decisional Conflict Scale.

Compared with the group receiving the ACR lupus pamphlet, participants who received the decision aid had less decisional conflict, as demonstrated by clinically meaningfully and statistically significantly larger reductions in the DCS post-intervention—a 21.8 (SE, 2.5)- versus a 12.7 (SE, 2.0)-point decrease (*p* = 0.005 from two-sample *t* test). Post-intervention decisional conflict scores and distribution are shown in **Fig 2**. After accounting for the amount of pre-intervention decisional conflict, the participants who received the decision aid had significantly less post-intervention decisional conflict compared with the pamphlet group, 11.5 (SE, 1.4) versus 24.8 (SE, 2.3) (*p* < 0.001 from ANCOVA). The proportion of patients with unresolved clinically significant decisional conflict (score ≥25) post-intervention was lower in the decision aid versus the pamphlet group, 22% versus 44% (*p* < 0.001 from ANCOVA). In the mediation analyses, we found that the reduction in decisional conflict was not mediated by the clarification of choices post-intervention (*p* = 0.298 for mediation by clarification of choices) or by improved knowledge (*p* = 0.063 for mediation by knowledge).

There was no difference in the informed choice regarding immunosuppressive medications in the main analysis, 41% in the decision aid versus 31% in pamphlet group (*p* = 0.08 from chi-squared test). There was also not a significant difference in informed choice after accounting for baseline knowledge, values, and choice (*p* = 0.10 from logistic regression). Using an alternate definition for patient values regarding immunosuppressive medications (a priori protocol specified [28]; sensitivity analysis), more women in the decision aid group made an informed choice compared with those in the pamphlet group, 50% versus 35% (*p* = 0.006). Using the test for superiority as per protocol [28], results were statistically significant for both main and sensitivity analyses (one-sided *p* = 0.04 and *p* = 0.003; **Table 2**; see statistical analysis section).

**Table 2:** Co-primary outcomes: DCS score and informed choice.

Outcomes	Decision Aid	Pamphlet	Difference between Treatment Arms		
	Mean (SEM) or *N* (%)	Mean (SEM) or *N* (%)	Odds Ratio (95% CI)	Mean Difference (95% CI)	*p*-value*
**Change in DCS total score**	21.8 (2.5)	12.7 (2.0)	N/A	9.1 (2.8–15.5)	0.005
**Change in DCS subscale scores**					
Change in Uncertainty subscale	17.3 (3.5)	5.0 (3.2)	N/A	12.2 (2.9–21.6)	0.01
Change in Informed subscale	30.6 (3.3)	21.7 (2.8)	N/A	8.9 (0.4–17.4)	0.04
Change in Values Clarity subscale	27.2 (3.4)	16.8 (3.1)	N/A	10.3 (1.3–19.4)	0.03
Change in Support subscale	12.4 (2.5)	6.1 (2.2)	N/A	6.4 (−0.2 to 12.9)	0.06
**Unresolved clinically significant decisional conflict on DCS (score ≥25)**	34 (22.5%)	65 (44.2%)	0.4 (0.2–0.6)	N/A	<0.001
**Informed choice for Immunosuppressives**			1.5 (1.0–2.5)	N/A	0.08
Informed	62 (41.1%)	46 (31.3%)			
Not informed	89 (58.9%)	101 (68.7%)			
**Informed choice components**					
Knowledge (percentage correct)	76.9 (1.0)	73.9 (1.1)	N/A	3.0 (0.1–5.9)	0.04
Values for immunosuppressives			0.8 (0.5–1.3)	N/A	0.34
Against	72 (47.7%)	62 (42.2%)			
In favor	79 (52.3%)	85 (57.8%)			
Choice for immunosuppressives			1.6 (0.9–2.7)*	N/A	0.10
Undecided	30 (19.9%)	41 (27.9%)			
Unwilling	11 (7.3%)	18 (12.2%)			
Willing	110 (72.9%)	88 (59.9%)			

*p-value was obtained from two-sample *t* tests (for continuous outcomes) or chi-squared tests (for categorical outcomes).

Abbreviations: CI, confidence interval; DCS, Decisional Conflict Scale; N/A, not applicable; SEM, standard error of the mean.

Compared with the provision of the ACR lupus pamphlet, decision aid use was associated with a statistically significant reduction in all DCS subscale scores (*p* < 0.05) except one: the feeling supported in decision-making subscale (*p* = 0.056; **Table 2**). Of the three informed choice components, compared with the participants in the ACR lupus pamphlet group, participants in the decision aid group had statistically significantly higher objective knowledge scores post-intervention (76.9 [SE, 1.0] versus 73 [SE, 1.1]; *p* = 0.045), and a clinically meaningful (≥10% absolute difference) but not statistically significantly higher proportion changed from unwilling or undecided pre-intervention to willing to take immunosuppressive medications post-intervention (47% versus 32%; *p* = 0.078).

**Fig 3:**
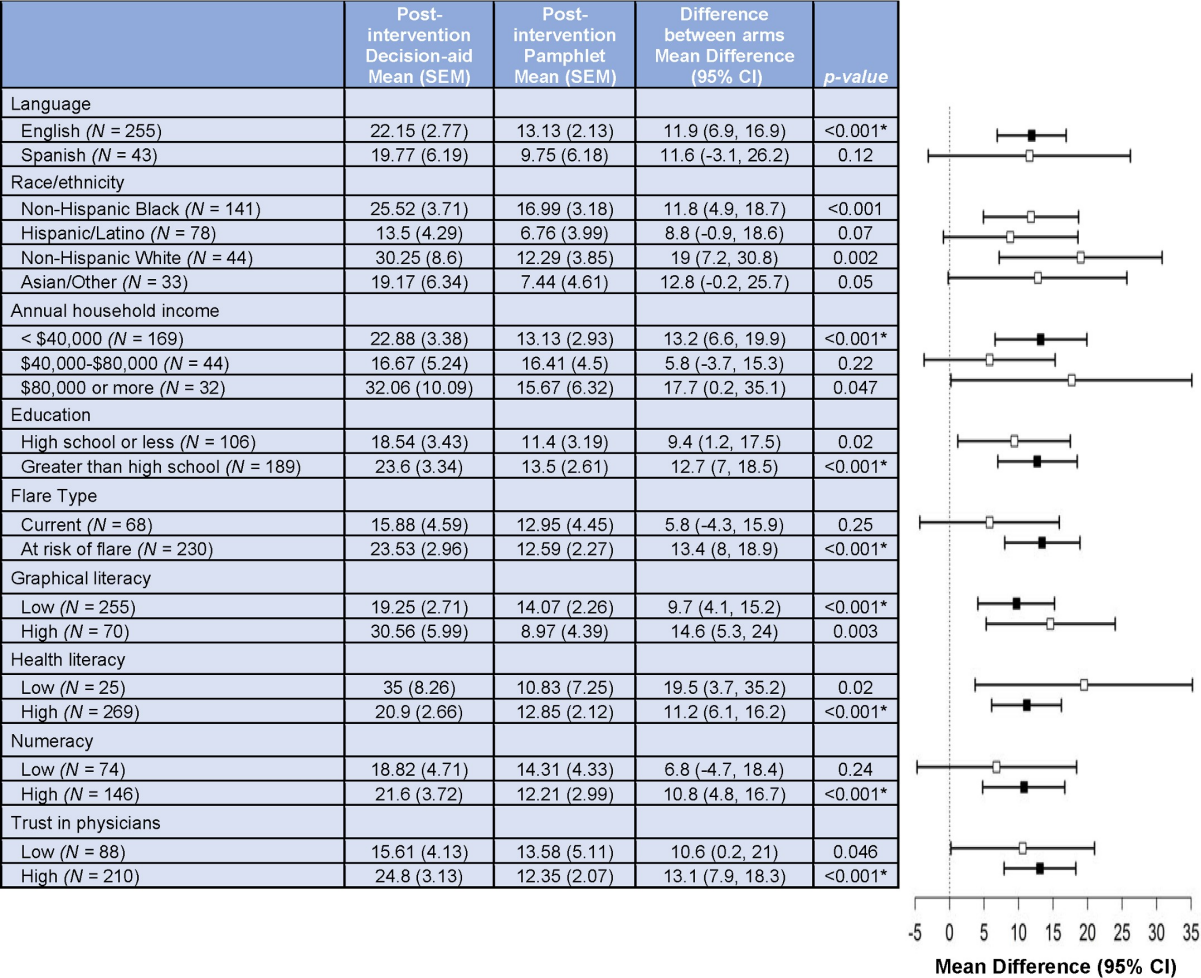
Subgroup analyses for decisional conflict scale (DCS) score. Differences that are statistically significant after correcting for multiple comparisons are represented with a filled square; others are presented with an open square. The hashed vertical line represents a difference of zero in DCS scores post-intervention, i.e., no difference between the decision aid and pamphlet groups. An asterisk (*) indicates statistically significant Bonferroni-corrected *p*-value (*p* < 0.0008). The categorization for subgroups were as follows: graphical literacy: low, 0–2, High, 3–4; SAHL: low, 0–14, high, >14; numeracy: low, 0–3, high, 4–6; trust in physicians: low, <44, high, 44–55. DCS, Decisional Conflict Scale; SAHL, Short Assessment of Health Literacy.

**Fig 4:**
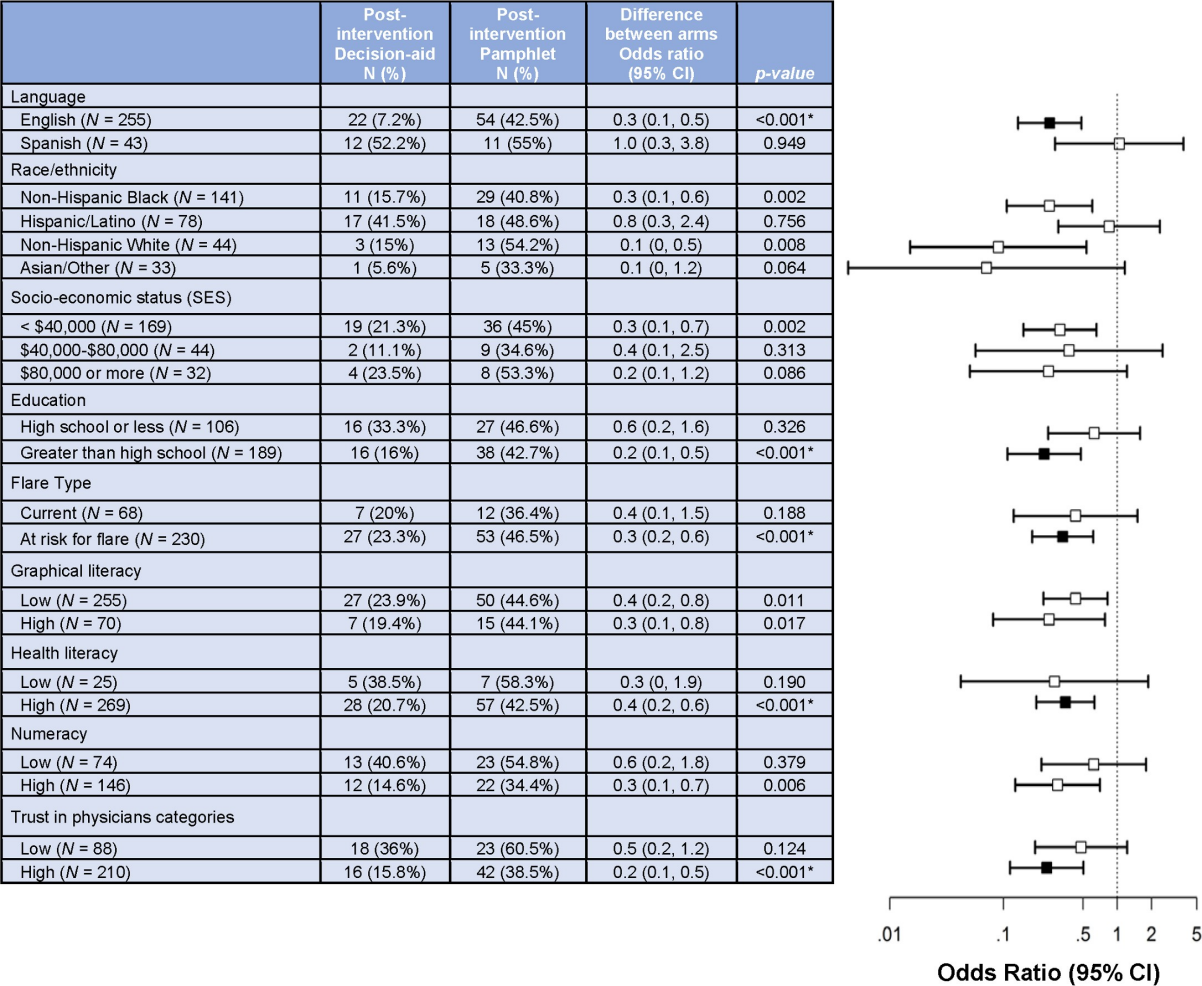
Subgroup analyses for unresolved decisional conflict scale (DCS) score of ≥ 25. Differences that are statistically significant after correcting for multiple comparisons are represented with a filled square; others are presented with an open square. The hashed vertical line represents an odds ratio of one for unresolved DCS scores post-intervention, i.e., no difference in the proportion of people with unresolved decisional conflict between the decision aid and the pamphlet groups. An asterisk (*) indicates statistically significant Bonferroni-corrected *p*-value (*p* < 0.0008). The categorizations for subgroups were as follows: graphical literacy: low, 0–2, high, 3–4; SAHL: low, 0–14; high, >14; numeracy: low, 0–3; high, 4–6; trust in physicians: low, <44, high, 44–55. DCS, Decisional Conflict Scale; SAHL, Short Assessment of Health Literacy.

In preplanned subgroup analyses, there was clinically meaningful and statistically significant reduction in the DCS score in the decision aid versus pamphlet group in patients with lower income and lower graphical literacy (**Fig 3**). A lower proportion had an unresolved clinically significant decisional conflict post-intervention in the decision aid versus pamphlet group in participants using the English (7% decision aid versus 43% pamphlet) versus the Spanish (52% versus 55%) language version (**Fig 4**; **S5 Text**). Respectively, a lower proportion of subjects in decision aid and pamphlet groups reported unresolved clinically significant decisional conflict post-intervention, with higher (21% versus 42%) versus lower (39% versus 58%) health literacy, with higher (16% versus 43%) versus lower education level (33% versus 47%), and more (16% versus 38%) versus less (36% versus 61%) trust in physicians (**Fig 4**; **S5 Text**). The remainder of subgroup analyses for informed choice were not significant based on Bonferroni-adjusted *p*-value (**S5 Text** and **S6 Text**).

### Secondary outcomes, acceptability, feasibility and adverse events

There were no statistically significant differences in any of the secondary outcomes in the decision aid versus pamphlet groups (higher score = better outcome for all), respectively: (1) IPC-SF mean (SD) scores were 83.6 (7.7) versus 83.1 (7.3) (*p* = 0.50; *n* = 296); (2) 94% versus 85% of patients had concordance in desired versus actual role played in decision-making using the control preferences scale (*p* = 0.25; *n* = 68; only in those with current flare); and (3) mean (SD) patient active participation score, 8.1 (7.2) versus 9.2 (7.3) (*p* = 0.80) in an analysis of audiotaped conversations (*n* = 33). Patient-centered communication by the doctor in audiotaped conversation showed a trend towards statistical significance, 5.1 (2.1) versus 3.7 (1.9) (*p* = 0.06). We found that 27% of the participants preferred cyclophosphamide as the treatment option, 33% other immunosuppressive medications (19% mycophenolate mofetil; 1% calcineurin inhibitors; 13% azathioprine), 29% were unsure of which treatment of the two treatment options they preferred, and 9% chose none (see **S4 Text** for treatment scenarios and choices; 2% missing).

More patients rated the decision aid versus pamphlet information as “excellent” for understanding lupus nephritis impact (49% versus 33%), risk factors (43% versus 27%), medication options (50% versus 33%), evidence about medications (47% versus 24%), and information about other patients (42% versus 22%) (*p* < 0.05 for all; **Table 3**). More patients in the decision aid versus pamphlet group agreed or strongly agreed that the decision aid was easy to use—51% versus 38% for the pamphlet (*p* = 0.006; **Table 3**). The majority (65%–90%) in both groups agreed strongly that study procedures including viewing of the decision aid or pamphlet (process, study questionnaires, extra time needed for the study) were feasible, with 65%–90% in both groups agreeing or strongly agreeing (**Table 3**).

**Table 3:** Acceptability and feasibility of lupus nephritis decision aid versus lupus pamphlet and feasibility of the study procedures.

Acceptability and Feasibility Assessments	Decision Aid	Pamphlet	*p*-value**
	(*n* = 151)	(*n* = 147)	
**Patient acceptability of information and presentation: Percentage of subjects marking "Excellent”**			
*Impact of lupus nephritis *	74 (49.0%)	49 (33.0%)	0.006
*Risk factors *	64 (42.4%)	40 (27.2%)	0.006
*Medication options*	76 (50.3%)	49 (33.3%)	0.003
*Evidence about medications*	71 (47.0%)	35 (23.8%)	<0.001
*Studies about others*	64 (42.4%)	32 (21.8%)	<0.001
**Feasibility of the study intervention**			
The education guide*was easy to use.			0.006
(Missing)	—	1 (0.7%)	
Strongly Disagree	1 (0.7%)	3 (2.0%)	
Disagree	1 (0.7%)	13 (8.8%)	
Neither Agree nor Disagree	73 (48.3%)	74 (50.3%)	
Agree	75 (49.7%)	55 (37.4%)	
Strongly Agree	1 (0.7%)	1 (0.7%)	
**Feasibility of the study procedures**			
*The questions were easy to see/hear*.			0.70
(Missing)	—	1 (0.7%)	
Strongly Disagree	1 (0.7%)	2 (1.4%)	
Disagree	5 (3.3%)	3 (2.0%)	
Neither Agree nor Disagree	5 (3.3%)	7 (4.8%)	
Agree	70 (46.4%)	75 (51.0%)	
Strongly Agree	70 (46.4%)	59 (40.1%)	
*The questions were easy to answer*.			0.10
(Missing)	—	1 (0.7%)	
Strongly Disagree	1 (0.7%)	3 (2.0%)	
Disagree	3 (2.0%)	9 (6.1%)	
Neither Agree nor Disagree	18 (11.9%)	27 (18.4%)	
Agree	73 (48.3%)	63 (42.9%)	
Strongly Agree	56 (37.1%)	44 (29.9%)	
*The process did not take too long*.			0.23
(Missing)	—	1 (0.7%)	
Strongly Disagree	4 (2.6%)	3 (2.0%)	
Disagree	25 (16.6%)	13 (8.8%)	
Neither Agree nor Disagree	22 (14.6%)	29 (19.7%)	
Agree	56 (37.1%)	63 (42.9%)	
Strongly Agree	44 (29.1%)	38 (25.8%)	
*I did not mind spending extra time at my doctor visit to understand the risks and benefits of treatment*.			0.23
(Missing)	—	1 (0.7%)	
Strongly Disagree	1 (0.7%)	4 (2.7%)	
Disagree	6 (4.0%)	1 (0.7%)	
Neither Agree nor Disagree	12 (7.9%)	13 (8.8%)	
Agree	63 (41.7%)	57 (38.8%)	
Strongly Agree	69 (45.7%)	71 (48.3%)	

*Education guide refers to the ACR pamphlet or the computerized, individualized decision aid.

**p-value using chi-squared test.

Each question is in italics.

Abbreviation: ACR, American College of Rheumatology.

One patient in each of the two intervention groups died: one at 53 days post-intervention, caused by right ventricular failure after cardiovascular surgery (decision aid), and one at 22 days post-intervention, caused by subarachnoid hemorrhage (pamphlet); both were unrelated to the study procedures or interventions. No other adverse events were reported.

## Discussion

In this multicenter RCT in 301 women with lupus kidney disease that included people of a racial/ethnic minority background and those with low SES, low literacy, or low income, an individualized, culturally tailored, computerized lupus nephritis decision aid for immunosuppressive medications (SMILE) was associated with a clinically meaningful and statistically significant reduction in decisional conflict compared with an ACR lupus information pamphlet (updated version at ACR website at https://www.rheumatology.org/I-Am-A/Patient-Caregiver/Diseases-Conditions/Lupus). A lower proportion in the decision aid group had unresolved clinically significant conflict. There was no statistical difference in the informed choice regarding immunosuppressive drugs between the decision aid and the pamphlet groups in the main analysis using the median patient value, but there was a clinically meaningful and statistically significant difference in the informed choice between groups in the prespecified sensitivity analysis that used net patient value (a clinical approach). In general, people are either favorable or not favorable towards immunosuppressive medications, which influences their decision-making and the final choice to use or not to use them for treatment. Therefore, the analysis considering the net patient value regarding the use of immunosuppressive medications (favoring versus against; a clinical approach) may be more clinically more meaningful than the analyses using a median value (a statistical approach). The number needed to treat to benefit (NNTB) with decision aid was 5 for a resolved decisional conflict (opposite of unresolved) and 7–10 for informed choice for immunosuppressive medication (sensitivity versus main analysis). The decision aid was tested in a population of women with lupus nephritis, in which the majority of the patients had low SES and many had low health literacy and numeracy or were non-English speaking. The lack of significance in certain subgroups (Hispanic women) is likely related to lack of power for this subgroup analysis, but might also indicate a lower efficacy; this needs to be explored in future studies. Even if a Bonferroni correction were applied because we had co-primary outcomes, the change in DCS would still be statistically significant at α = 0.025 (= 0.05/2) and the difference in informed choice in main analysis would remain nonsignificant.

A large-scale implementation trial of this self-administered computerized decision aid (SMILE) in 16 busy U.S. clinical practices has been recently funded by PCORI and is underway. SMILE will be available free of cost in the public domain and can be administered using any touchscreen computer.

In a Cochrane systematic review of 105 studies, the use of the decision aid was associated with people being more knowledgeable, better informed, and clearer about their values and they played a more active role in their treatment with moderate- to high-quality evidence [10]. The magnitude of the effect on decisional conflict was similar in participants with low versus high education level, health literacy, or graphical literacy. Racial/ethnic minorities and individuals with lupus with limited socioeconomic resources have barriers to optimal treatment [22–24]. Decision aids have succeeded in improving outcomes in other conditions, when developed for the target population [58], similar to ours. We believe that the use of the lupus nephritis decision aid can play a role in improving outcomes of the group of patients with lupus nephritis.

This study focused on women from racial/ethnic minorities, with a majority with low SES and low graphical literacy, and many with low health literacy and numeracy skills, or who were non-English speaking. To our knowledge, this is the first study to provide data supporting the efficacy of an individualized, culturally tailored, computerized decision aid in lupus nephritis. In a previous study, a lupus decision board was developed to improve the quality of time spent in medical consultations [59], but no data on further development of a decision aid were published.

In a related field of behavioral interventions involving medication adherence in lupus [60], pharmacist-led counseling was associated with improved adherence compared with physician counseling [61], while mobile text messaging with reminders did not improve medication adherence [62]. To our knowledge, no lupus decision aids are available, and none of the educational or behavioral interventions in lupus have previously been tested in a high-risk patient population with low SES, low health literacy and numeracy, and/or non-English–speaking patients, in contrast to our study.

We incorporated formative work [22–24], state-of-the-art CER [25–27], and multi-stakeholder input to develop an individualized, computerized, patient decision aid tailored to women with lupus nephritis facing a critical medication decision. Few decision aids have been developed to support decision-making for an analogous high-risk patient population, i.e., African-Americans, low SES, and low graphical and health literacy. We believe that this tool, available in English and Spanish, can facilitate and improve shared decision-making for lupus nephritis treatments in clinical practice and that it should lead to higher patient satisfaction and engagement. Whether it will improve disease outcomes over time will require further study. The tool will be available free of charge in the public domain and can be self-administered using touchscreen computers.

Our study has some limitations. Study findings are not generalizable to men, because men were not involved in the tool’s development or testing. This decision aid is specific to lupus nephritis, as it is the most common and most well studied of all organ-threatening lupus manifestations, for which multiple treatments are available. It can be adapted to develop a tool specific for other lupus manifestations with minor modifications, because the treatments for other lupus manifestations are similar to that of lupus nephritis. The effect of decision aid on long-term lupus outcomes was not tested but needs to be tested in future studies. Translations into other languages are needed, and the inclusion of a larger number of Asian and Native American women in future testing is needed. This will further improve the generalizability of this tool to all women with lupus nephritis. The decision aid was self-administered to patients in the clinic waiting room; whether it can be adapted for at-home use by patients before a clinic visit is unknown, and needs to be tested. Assessment of exploratory outcomes, including renal function, proteinuria, etc., was not possible due to heterogeneity of performance of these measures in routine clinical care across four sites, limited study resources, and the inclusion of fewer patients with current lupus nephritis flare than anticipated. Our patient decision aid, SMILE, was administered on a tablet computer compared with the control intervention, a paper pamphlet, which represents two different methods of intervention. The method of intervention may have contributed to the success of our intervention.

Study strengths include that we sought input from a wide range of patients of all race/ethnicities, including Spanish-speaking patients, and adhered to IPDAS principles for decision aid development [35]. Our study was a multicenter study with geographical diversity; included vulnerable populations who are at risk for poor outcomes, including those from racial/ethnic minority groups, with low educational level, income, health, and graphical literacy; and designated the standard of care (ACR lupus paper pamphlet) as the attention control. High ratings on the acceptability and feasibility of content and presentation indicated that our tool is user-friendly.

In a diverse group of women with lupus nephritis, including those with low educational level, income, health literacy, or graphical literacy, an individualized, culturally tailored, computerized self-administered patient decision aid (SMILE) administered in clinic waiting rooms was more effective than the usual practice (standard ACR paper pamphlet) for immunosuppressive medications decision-making. Large, multicenter trials are needed to establish the generalizability of this benefit. In collaboration with our partners, the Lupus Foundation of America (LFA) and the Arthritis Foundation (AF), further research is planned to understand the best way to implement this tool in busy clinical practices and to widely disseminate this decision aid to patients (e.g., smartphone application).

## Supporting information

S1 CONSORT Checklist CONSORT 2010 checklist of information to include when reporting a randomized trial. (DOC)

S1 Text The ACR lupus pamphlet: English and Spanish language versions. ACR, American College of Rheumatology.
(DOCX)

S2 Text Study inclusion and exclusion criteria. (DOCX)

S3 Text Primary and secondary outcome measures for RCT. RCT, randomized controlled trial.
(DOCX)

S4 Text Detailed methods: Treatment scenarios for lupus decision aid and details for calculation of informed choice. (DOCX)

S5 Text Subgroup analyses for informed choice, main analysis (using the median value, i.e., statistical approach)*. An asterisk (*) indicates that one subject was excluded due to missing pre-intervention informed choice. A double asterisk (**) indicates that no subgroup differences were statistically significant at the Bonferroni-corrected *p*-value (*p* < 0.0008). Graphical literacy: low, 0–2; high, 3–4. SAHL: low, 0–14; high, >14. Numeracy: low, 0–3; high, 4–6. Trust in physicians: low, <44; high, 44–55. SAHL, Short Assessment of Health Literacy.
(DOCX)

S6 Text Subgroup analyses for informed choice, sensitivity analyses (using the net positive or negative value, i.e., clinical approach)*. An asterisk (*) indicates that one subject was excluded due to missing pre-intervention informed choice. A double asterisk (**) indicates that no subgroup differences were statistically significant at the Bonferroni-corrected *p*-value (*p* < 0.0008). Graphical literacy: low, 0–2; high, 3–4. SAHL: low, 0–14; high, >14. Numeracy: low, 0–3; high, 4–6. Trust in physicians: low, <44; high, 44–55. SAHL, Short Assessment of Health Literacy.
(DOCX)

S1 Fig Screenshots of individualized, culturally appropriate, computerized decision aid for lupus nephritis called SMILE. SMILE, shared decision-making in lupus electronic tool.
(TIFF)

## Abbreviations

ACR: American College of Rheumatology
AF: Arthritis Foundation
APPC: Active Patient Participation Coding Scheme
CER: comparative effectiveness research
DCS: Decisional Conflict Scale
EHR: electronic health record
ESRD: end-stage renal disease
HIPAA: Health Insurance Portability and Accountability Act
IDEA-WON: Individualized Decision aid for Diverse Women with Lupus Nephritis
IPC-SF: Interpersonal Processes of Care short form
IPDAS: International Patient Decision aid Standards
LFA: Lupus Foundation of America
NNTB: number needed to treat to benefit
PCORI: Patient-Centered Outcomes Research Institute
RCT: randomized controlled trial
SAHL: Short Assessment of Health Literacy
SD: standard deviation
SE: standard error
SES: socioeconomic status
SLE: systemic lupus erythematosus
SMILE: shared decision-making in lupus electronic tool
UAB: University of Alabama at Birmingham
UCSF: University of California at San Francisco
