# Supplementary material for: Individualized decision aid for diverse women with lupus nephritis (IDEA-WON): A randomized controlled trial
Source: PLoS Med. 2019 May 8;16(5):e1002800. doi: 10.1371/journal.pmed.1002800 (PMC6505936; doi:10.1371/journal.pmed.1002800)
Supplement: S1 Text — ACR, American College of Rheumatology. (DOCX) [file pmed.1002800.s002.docx]

## S1 text. The American College of Rheumatology (ACR) lupus pamphlet: English and Spanish language versions


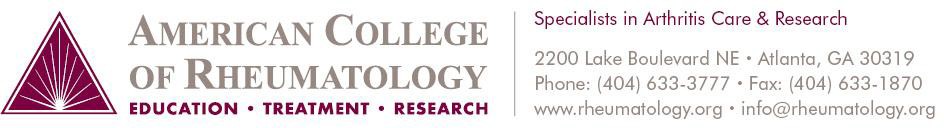


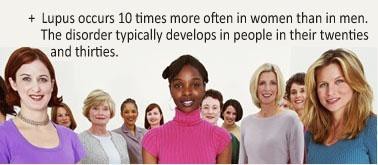


**Systemic Lupus Erythematosus (Lupus)**

Systemic lupus erythematosus, referred to as SLE or lupus, is sometimes called the “great imitator.” Why? Because of its wide range of symptoms, people often confuse lupus with other health problems.

Lupus affects mainly the joints, kidneys and skin. It can range from mild to serious. Yet, there is much reason for hope. Improvements in treatment have greatly improved these patients’ quality of life and increased their lifespan.

## Fast facts


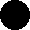
Lupus affects 10 times as many women as men.


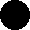
Treatment depends on the symptoms and how serious they are.


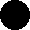
Because it is a complex disease, lupus requires treatment by or consultation with a rheumatologist, a doctor who is an expert in treating diseases like lupus.


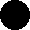
People can live well with lupus if they actively work toward good health.

## What is lupus?

Lupus is a chronic (long-term) disease that causes inflammation — pain and swelling. It can affect the skin, joints, kidneys, lungs, nervous system and other organs of the body. Most patients feel fatigue and have rashes, arthritis (painful and swollen joints) and fever.

Lupus flares vary from mild to serious. Most patients have times when the disease is active, followed by times when the disease is mostly quiet — referred to as a remission.

## What causes lupus?

The immune system is the body’s defense system. When healthy, it protects the body by making antibodies (blood proteins) that attack foreign germs and cancers. With lupus, the immune system misfires. Instead of producing protective antibodies, an autoimmune disease begins and makes “autoantibodies,” which attack the patient’s own tissues. Doctors sometimes refer to this as a “loss of self-tolerance.”

As the attack goes on, other immune cells join the fight. This leads to inflammation and abnormal blood vessels ([vasculitis](http://www.rheumatology.org/practice/clinical/patients/diseases_and_conditions/vasculitis.asp)). These antibodies then end up in cells in organs, where they damage those tissues.

Why this inflammatory response begins is not clear. It most likely results from a mix of inherited tendencies and things in your environment. These include viruses, sunlight and drug allergies. People with lupus may also have an impaired process for clearing old and damaged cells from the body, which causes an abnormal immune response.

## Who gets lupus?

Most often, lupus starts in people in their 20s and 30s. It occurs 10 times more often in women than in men. The disease is more common in some ethnic groups, mainly blacks and Asians, and tends to be worse in these groups.

## How is lupus diagnosed?


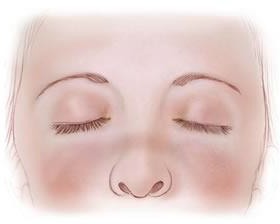
Lupus can be hard to detect because it has many symptoms, and they can come on slowly.

**Symptoms.** People with lupus often have features that are not specific to lupus. These include fever, fatigue, weight loss, blood clots and hair loss in spots or around the hairline. They may also have heartburn, stomach pain, and poor circulation to the fingers and toes. Pregnant women can have miscarriages.

The American College of Rheumatology has a list of symptoms and other measures that doctors can use as a guide to decide if a patient with symptoms has lupus. If your doctor finds that you have at least four of these problems, and finds no other reason for them, you may have lupus:

This picture shows a malar rash, a red rash on the cheeks and nose. This rash often results from sun exposure.

##
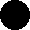
Rashes:


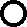

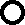

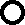
butterfly-shaped rash over the cheeks — referred to as malar rash red rash with raised round or oval patches — known as discoid rash rash on skin exposed to the sun


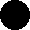
**Mouth sores:** sores in the mouth or nose lasting from a few days to more than a month


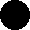
**Arthritis:** tenderness and swelling lasting for a few weeks in two or more joints


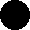

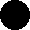

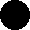
**Lung or heart inflammation:** swelling of the tissue lining the lungs (referred to as pleurisy or pleuritis) or the heart (pericarditis), which can cause chest pain when breathing deeply **Kidney problem:** blood or protein in the urine, or tests that suggest poor kidney function **Neurologic problem:** seizures, strokes or psychosis (a mental health problem)

##
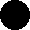
Abnormal blood tests:


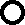
low blood cell counts: anemia, low white blood cells or low platelets


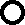
positive [antinuclear antibody](http://www.rheumatology.org/practice/clinical/patients/diseases_and_conditions/ana.asp): referred to as ANA and present in nearly all patients with lupus


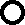
certain antibodies that show an immune system problem: anti-double-strand DNA (called anti-dsDNA), anti-Smith (referred to as anti-Sm) or antiphospholipid antibodies, or a false-positive blood test for syphilis (meaning you do not really have this infection)

**Lab tests.** If your doctor suspects you have lupus from your symptoms, you will need a series of blood tests to confirm that you do have the disease. The most important blood screening test measures [ANA](http://www.rheumatology.org/practice/clinical/patients/diseases_and_conditions/ana.asp), but you can have ANA and not have lupus. Therefore, if you have positive ANA, you may need more specific tests to prove the diagnosis. These blood tests include antibodies to anti-dsDNA and anti-Sm.

The presence of antiphospholipid antibodies can help doctors detect lupus. These antibodies signal a raised risk of certain complications such as miscarriage, difficulties with memory, or blood clots that may lead to stroke or lung injury. Doctors also may measure levels of certain complement proteins (a part of the immune system) in the blood, to help detect the disease and follow its progress.

## How is lupus treated?


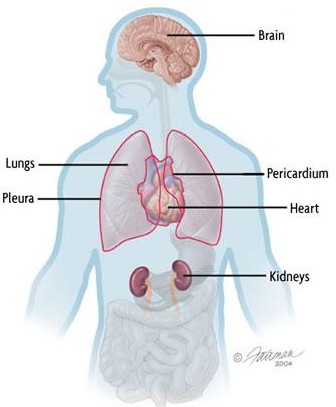
There is no cure for lupus, and treatment can be a challenge. However, treatment has improved a great deal.

Treatment depends on the type of symptoms you have and how serious they are. Patients with muscle or joint pain, fatigue, rashes and other problems that are not dangerous can receive “conservative” treatment. These options include nonsteroidal anti-inflammatory drugs — referred to as [NSAIDs](http://www.rheumatology.org/practice/clinical/patients/medications/nsaids.asp).

**Nonsteroidal anti-inflammatory drugs.** NSAIDs decrease swelling, pain and fever. These drugs include ibuprofen (brand names Motrin, Advil) and naproxen (Naprosyn, Aleve). Some of these NSAIDs can cause serious side effects like stomach bleeding or kidney damage. Always check with your doctor before taking any medications that are over the counter (without a prescription) for your lupus*.*

Lupus is a disease that can lead to inflammation in not just the joints and skin but also many other organs. These include the kidneys, the tissue lining the lungs (pleura) and heart (pericardium), and the brain.

**Antimalarial drugs.** Patients with lupus also may receive an antimalarial medication such as [hydroxychloroquine](http://www.rheumatology.org/practice/clinical/patients/medications/hydroxychloroquine.asp) (Plaquenil). Though these drugs prevent and treat malaria, they also help relieve some lupus symptoms, such as fatigue, rashes, joint pain or mouth sores. They also may help prevent abnormal blood clotting.

**Corticosteroids and immune suppressants.** Patients with serious or life-threatening problems such as kidney inflammation, lung or heart involvement, and central nervous system symptoms need more “aggressive” (stronger) treatment. This may include high-dose corticosteroids such as prednisone (Deltasone and others) and drugs that suppress the immune system. Immune suppressants include [azathioprine](http://www.rheumatology.org/practice/clinical/patients/medications/azathioprine.asp) (Imuran), [cyclophosphamide](http://www.rheumatology.org/practice/clinical/patients/medications/cyclophosphamide.asp) (Cytoxan) and [cyclosporine](http://www.rheumatology.org/practice/clinical/patients/medications/cyclosporine.asp) (Neoral, Sandimmune)*.* Recently [mycophenolate mofetil](http://www.rheumatology.org/practice/clinical/patients/medications/mycophenolate.asp) (CellCept) has been used to treat severe kidney disease in lupus—referred to as lupus nephritis.

**Biologics.** New treatment options include drugs called biologics that are already approved for treatment of other rheumatic diseases such as rheumatoid arthritis. Examples are rituximab (Rituxan) and abatacept (Orencia). These two drugs are not approved for treatment of lupus. In 2011, though, the FDA approved a biologic, belimumab (Benlysta), for treatment of mild to moderate (medium severe) SLE. It is the first new drug approved for lupus since 1955.

This exciting treatment advance occurred thanks to research studies in patients—called [clinical trials](http://www.rheumatology.org/practice/clinical/patients/diseases_and_conditions/clinicalresearchtrials.asp). It provides hope that some of the other drugs that researchers are testing in patients will help lupus. It also underscores the need for patients with lupus to take part in studies.

**Combination treatment.** Health care providers may combine a few medications to control lupus and prevent tissue damage.

Each treatment has risks and benefits. Most immune-suppressing medications, for instance, may cause major side effects. Side effects of these drugs may include a raised risk of infections as well as nausea, vomiting, hair loss, diarrhea, high blood pressure and osteoporosis (weak bones). Rheumatologists may lower the dose of a drug or stop a medicine because of side effects or when the disease goes into remission. As a result, it is important to receive careful and frequent health exams and lab tests to track your symptoms and change your treatment as needed.

## Broader health impact of lupus

Even when it is not active, lupus may cause problems later. Some of these problems can be fatal. One of these problems is atherosclerosis (clogging of the arteries) that may develop in younger women or may be more severe than usual. This problem raises the risk of heart attacks, heart failure and strokes. Thus, it is vital that patients with lupus lower their other risk factors for heart disease, such as smoking, high blood pressure and high cholesterol. It is also important to have as active a lifestyle as possible.

Lupus may also cause kidney disease, which can advance to kidney failure and need dialysis. You can help prevent these serious problems by seeking treatment at the first signs of kidney disease. These signs include:


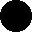

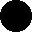

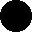
High blood pressure Swollen feet and hands Puffiness around your eyes


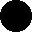
Changes in urination (blood or foam in the urine, going to the bathroom more often at night, or pain or trouble urinating)

## Living with lupus

Treatment of lupus has improved, and people with the disease are living longer. But, it is still a chronic disease that can limit activities. Quality of life can suffer because of symptoms like fatigue and joint pain. Furthermore, some people do not respond to some treatments. Also, you may not be able to predict when lupus will flare. Such problems can lead to depression, anger, loss of hope or loss of the will to keep fighting.

The best way to control lupus is to follow these tips.


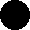
**Form a support system.** A good doctor-patient relationship and support from family and friends can help you cope with this chronic and often unpredictable illness.


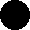
**Get involved in your care.** Take all medications as your doctor prescribed, and visit your doctor often. Learn as much as you can about lupus and your medications, and what kind of progress to expect.


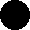
**Stay active.** Exercise helps keep joints flexible and may prevent heart disease and strokes. This does not mean overdoing it. Switch off doing light to moderate exercise with times of rest.


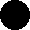
**Avoid excess sun exposure.** Sunlight can cause a lupus rash to flare and may even trigger a serious flare of the disease itself. When outdoors on a sunny day, wear protective clothing (long sleeves, a big-brimmed hat) and use lots of sunscreen.

**Pregnancy.** If you are a young woman with lupus and wish to have a baby, carefully plan your pregnancy. With your doctor’s guidance, time your pregnancy for when your lupus activity is low. While pregnant, avoid medications that can harm your baby. These include [cyclophosphamide](http://www.rheumatology.org/practice/clinical/patients/medications/cyclophosphamide.asp), [cyclosporine](http://www.rheumatology.org/practice/clinical/patients/medications/cyclosporine.asp), and [mycophenolate mofetil.](http://www.rheumatology.org/practice/clinical/patients/medications/mycophenolate.asp) If you must take any of these medicines, or your disease is very active, use birth control. For more information, see “[Pregnancy and Rheumatic Disease](http://www.rheumatology.org/practice/clinical/patients/diseases_and_conditions/pregnancy.asp).”

**Estrogen.** Rheumatologists have long been concerned that the female hormone estrogen or treatment with estrogen may cause or worsen lupus. Recent research showed that estrogen therapy can trigger some mild or moderate flares of lupus, but does not cause symptoms to get much worse. Yet, estrogen can raise the risk of blood clots. Thus, you should not take estrogen if your blood tests show antiphospholipid antibodies (meaning you already have a high risk of blood clots).

## Points to remember

Most people with lupus can live normal lives. To prevent serious problems, you should see your rheumatologist often. This lets your doctor keep track of your disease and change your treatment as needed.

If you do not live near a rheumatologist, you may need to have your primary care doctor manage your lupus with the help of a rheumatologist.

## The role of a rheumatologist in the treatment of lupus


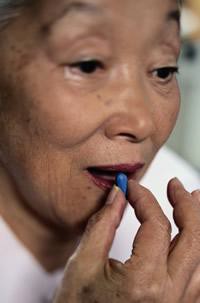


The best way to control lupus is to get involved in your care. Take all medications as your doctor prescribed, and visit your physician often. Learn as much as you can about lupus and your medications.

Lupus is a complex disease. As experts in diagnosing and treating autoimmune diseases such as lupus, rheumatologists can best advise patients about treatment options.

## To find a rheumatologist

For a listing of rheumatologists in your area, [click here.](http://www.rheumatology.org/directory/geo.asp?aud=pat)

Learn more about [rheumatologists](http://www.rheumatology.org/practice/clinical/patients/rheumatologist.asp) and [rheumatology health](http://www.rheumatology.org/practice/clinical/patients/healthprofessionals/index.asp) [professionals.](http://www.rheumatology.org/practice/clinical/patients/healthprofessionals/index.asp)

## For more information

The American College of Rheumatology has compiled this list to give you a starting point for your own additional research. The ACR does not endorse or maintain these websites, and is not responsible for any information or claims provided on them. It is always best to talk with your rheumatologist for more information and before making any decisions about your care.

The Arthritis Foundation [www.arthritis.org](http://www.arthritis.org/)

The ACR's Lupus Initiative [www.thelupusinitiative.org](http://www.thelupusinitiative.org/)

The Lupus Foundation of America [www.lupus.org](http://www.lupus.org/)

Lupus Research Institute [www.LupusResearchInstitute.org](http://www.lupusresearchinstitute.org/)

National Institute of Arthritis and Musculoskeletal and Skin Diseases [www.niams.nih.gov/Health_Info/Lupus/default.asp](http://www.niams.nih.gov/Health_Info/Lupus/default.asp)

**Updated February 2013**

Written by Ellen Ginzler, MD, and Jean Tayar, MD, and reviewed by the American College of Rheumatology Communications and Marketing Committee.

*This patient fact sheet is provided for general education only. Individuals should consult a qualified health care provider for professional medical advice, diagnosis and treatment of a medical or health condition.*

© 2013 American College of Rheumatology


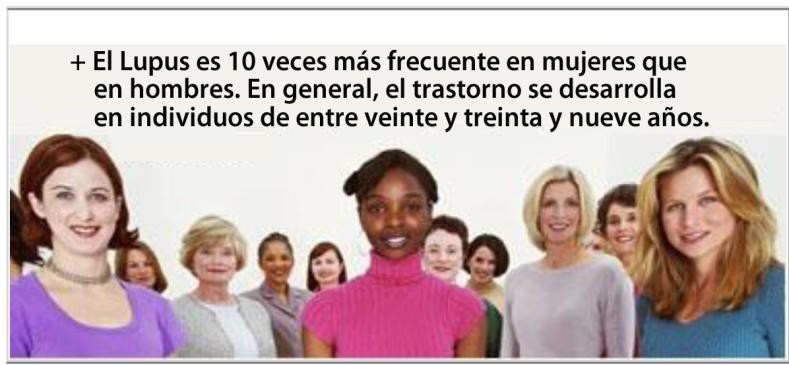


# Lupus eritematoso sistémico (lupus)

Al lupus eritematoso sistémico, conocido como SLE o lupus, en ocasiones se lo denomina "el gran imitador". ¿Por qué? Debido a su amplia variedad de síntomas, las personas a menudo confunden el lupus con otros problemas de salud.

El lupus afecta principalmente a las articulaciones, los riñones y la piel. Puede variar de leve a grave. Sin embargo, hay muchos motivos para tener esperanzas. Las mejoras en el tratamiento han optimizado notablemente la calidad de vida de estos pacientes y han incrementado su tiempo de vida.

## Datos breves


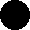

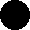
El lupus afecta 10 veces más a mujeres que hombres. El tratamiento depende de los síntomas y su gravedad.


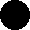
Debido a que es una enfermedad compleja, el lupus requiere un tratamiento por parte de un reumatólogo o una consulta con este, ya que dicho profesional es un médico experto en el tratamiento de enfermedades como el lupus.


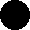
Las personas pueden vivir bien con lupus si trabajan activamente para lograr un buen estado de salud.

## ¿Qué es el lupus?

El lupus es una enfermedad crónica (de larga duración) que causa inflamación: dolor e hinchazón. Puede afectar la piel, las articulaciones, los riñones, los pulmones, el sistema nervioso y otros órganos del cuerpo. La mayoría de los pacientes sienten fatiga y tienen erupciones, artritis (dolor e hinchazón en las articulaciones) y fiebre.

Los brotes de lupus varían de leves a graves. La mayoría de los pacientes tienen momentos en que la enfermedad está activa, seguidos de otros en que principalmente se apacigua: esto se conoce como remisión.

## ¿Qué causa el lupus?

El sistema inmunológico es el sistema de defensa del organismo. Cuando está sano, el organismo produce anticuerpos (proteínas de la sangre) que atacan gérmenes extraños y cánceres con el fin de proteger el cuerpo. El lupus impide que el sistema inmunológico logre su objetivo. En lugar de producir anticuerpos protectores, se produce una enfermedad autoinmune que crea "autoanticuerpos" que atacan los propios tejidos del paciente. A veces los médicos lo llaman "pérdida de autotolerancia".

A medida que el ataque continúa, otras células del sistema inmunológico se unen a la lucha. Esto genera inflamación y vasos sanguíneos anormales ( [vasculitis](http://www.rheumatology.org/practice/clinical/patients/diseases_and_conditions/vasculitis.asp) ). Estos anticuerpos terminan luego en las células de los órganos, donde dañan dichos tejidos.

No está claro por qué se inicia esta respuesta inflamatoria. Lo más probable es que sea el resultado de una mezcla de tendencias heredadas y elementos del entorno. Estos incluyen los virus, la luz del sol y las alergias a medicamentos. Las personas con lupus también pueden presentar deficiencias en el proceso de limpieza de las células viejas y dañadas del cuerpo, lo que provoca una respuesta anormal del sistema inmunológico.

## ¿Quién contrae lupus?

Muy a menudo, el lupus comienza en personas de entre 20 y 40 años. Es 10 veces más frecuente en mujeres que en hombres. La enfermedad es más común en algunos grupos étnicos, principalmente individuos negros y asiáticos, y tiende a ser peor en estos grupos.

## ¿Cómo se diagnostica el lupus?


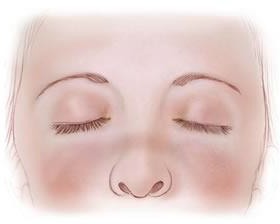
El lupus puede ser difícil de detectar porque presenta muchos síntomas que pueden aparecer lentamente.

**Síntomas.** Las personas con lupus suelen presentar características que no son específicas del lupus. Estas incluyen fiebre, fatiga, pérdida de peso, coágulos de sangre y pérdida de cabello en lugares determinados o alrededor del nacimiento del cabello. También pueden presentar ardor de estómago, dolor de estómago y mala circulación en los dedos de las manos y los pies. Las mujeres embarazadas pueden sufrir abortos espontáneos.

Esta imagen muestra un eritema malar, una erupción roja en las mejillas y la nariz. Esta erupción a menudo deriva de la exposición al sol.

El Colegio Estadounidense de Reumatología (ACR, por sus siglas en inglés) cuenta con una lista de síntomas y otras medidas que los médicos pueden usar como guía para decidir

si un paciente con síntomas padece de lupus. Si su médico descubre que usted sufre de al menos cuatro de estos problemas y no encuentra otro motivo relacionado con ellos, es posible que padezca de lupus:

##
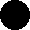
Erupciones:


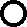

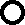
erupción con forma de mariposa en las mejillas, conocida como eritema malar erupción de color rojo con zonas ovaladas o redondos elevadas, conocida como erupción discoide


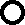
erupción en la piel expuesta al sol


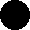
**Llagas en la boca:** llagas en la boca o la nariz que permanecen durante algunos días o incluso más de un mes


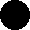
**Artritis:** sensibilidad e inflamación que dura algunas semanas en dos o más articulaciones


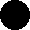
**Inflamación pulmonar o del corazón:** inflamación del tejido que recubre los pulmones (conocido como pleuresía o pleuritis) o el corazón (pericarditis), que puede causar dolor en el pecho al respirar profundamente


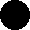

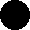

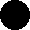
**Problema renal:** sangre o proteína en la orina, o exámenes que sugieren una función renal deficiente **Problema neurológico:** convulsiones, derrames cerebrales o psicosis (problema de salud mental) **Exámenes de sangre anormales:**


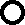
escasa cantidad de glóbulos rojos: anemia, escasa cantidad de glóbulos blancos o plaquetas


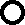
 [anticuerpos antinucleares](http://www.rheumatology.org/practice/clinical/patients/diseases_and_conditions/ana.asp) positivos: conocidos como ANA, están presentes en casi todos los pacientes con lupus


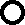
ciertos anticuerpos que muestran un problema del sistema inmunológico: antiADN de doble cadena (llamados antidsADN), antiSmith (conocidos como antiSm) o anticuerpos antifosfolípidos, o exámenes de sangre para detectar sífilis con resultado falso positivo (lo que significa que, en realidad, no tiene esta infección)

**Exámenes de laboratorio.** Si su médico sospecha que usted tiene lupus en función de los síntomas, deberá hacerse una serie de exámenes de sangre para confirmar que realmente padece esta enfermedad. El examen de sangre de control más importante mide los [ANA ,](http://www.rheumatology.org/practice/clinical/patients/diseases_and_conditions/ana.asp) pero usted puede tener ANA y no tener lupus. Por lo tanto, si tiene ANA positivos, es posible que necesite exámenes más específicos para confirmar el diagnóstico. Estos exámenes de sangre incluyen anticuerpos para antidsADN y antiSm.

La presencia de anticuerpos antifosfolípidos puede ayudar a los médicos a detectar el lupus. Estos anticuerpos indican un aumento del riesgo de ciertas complicaciones, tales como aborto espontáneo, dificultades con la memoria o coágulos sanguíneos que pueden provocar un accidente cerebrovascular o una lesión pulmonar. Los médicos también pueden medir los niveles de determinadas proteínas del complemento (una parte del sistema inmunológico) en la sangre para ayudar a detectar la enfermedad y seguir su desarrollo.

##
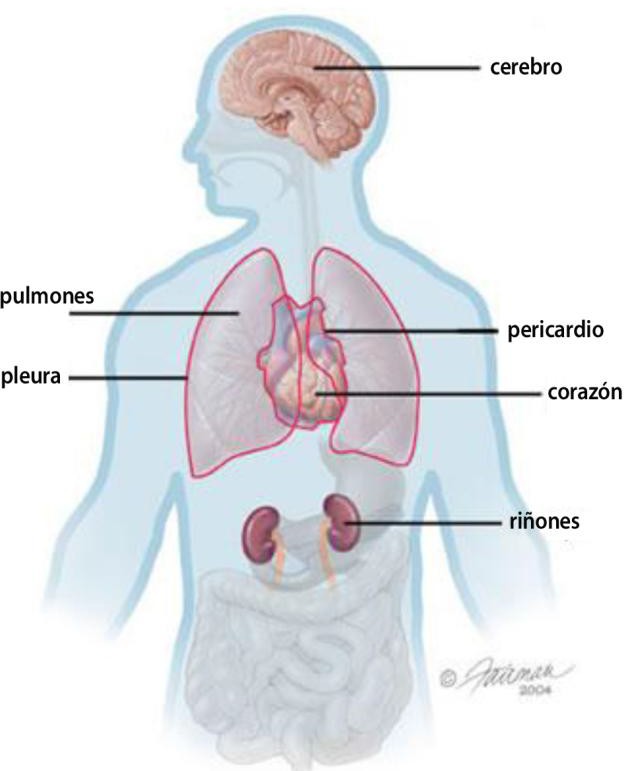
¿Cómo se trata el lupus?

No hay cura para el lupus, y el tratamiento puede resultar un desafío. Sin embargo, el tratamiento ha mejorado mucho.

Dicho tratamiento depende del tipo de síntomas que tiene y de su gravedad. Los pacientes con dolor muscular o articular, fatiga, erupciones y otros problemas que no son peligrosos pueden recibir un tratamiento "conservador". Estas opciones incluyen medicamentos antiinflamatorios no esteroideos - conocidos como [NSAID](http://www.rheumatology.org/practice/clinical/patients/medications/nsaids.asp) .

**Medicamentos antiinflamatorios no esteroideos.** Los NSAID disminuyen la hinchazón, el dolor y la fiebre. Estos medicamentos incluyen ibuprofeno (nombres comerciales: Motrin, Advil) y naproxeno (Naprosyn, Aleve) . Algunos de estos NSAID pueden causar efectos secundarios graves, como sangrado en el estómago o daño en el riñón. Siempre consulte con su médico antes de tomar cualquier medicamento de venta libre (sin receta) para el tratamiento del lupus*.*

El lupus es una enfermedad que puede derivar en inflamación, no sólo en las articulaciones y la piel, sino también en muchos otros órganos. Estos incluyen los riñones, el tejido que recubre los pulmones (pleura), el corazón (pericardio) y el cerebro.

**Medicamentos contra la malaria.** Los pacientes con lupus también pueden recibir un medicamento contra la malaria como [hidroxicloroquina](http://www.rheumatology.org/practice/clinical/patients/medications/hydroxychloroquine.asp)(Plaquenil). Aunque estos medicamentos previenen y tratan la malaria, también ayudan a aliviar algunos síntomas del lupus, tales como fatiga, erupciones, dolor en las articulaciones o llagas en la boca. También pueden ayudar a prevenir la coagulación sanguínea anormal.

**Corticosteroides e inmunosupresores.** Los pacientes con problemas graves o que suponen riesgo de vida, tales como inflamación en los riñones, compromiso pulmonar o cardíaco y síntomas relacionados con el sistema nervioso central, necesitan un tratamiento más "agresivo" (más fuerte). Esto puede incluir altas dosis de corticosteroides, como prednisona (Deltasone y otros) y medicamentos que suprimen el sistema inmunológico. Los inmunosupresores incluyen [azatioprina](http://www.rheumatology.org/practice/clinical/patients/medications/azathioprine.asp)(Imuran), [ciclofosfamida](http://www.rheumatology.org/practice/clinical/patients/medications/cyclophosphamide.asp)(Cytoxan)y [ciclosporina](http://www.rheumatology.org/practice/clinical/patients/medications/cyclosporine.asp)(Neoral, Sandimmune)*.* Recientemente se ha utilizado [micofenolato mofetil](http://www.rheumatology.org/practice/clinical/patients/medications/mycophenolate.asp) (CellCept) para tratar una enfermedad renal grave derivada del lupus, conocida como nefritis por lupus.

**Medicamentos biológicos.** Las nuevas opciones de tratamiento incluyen medicamentos llamados biológicos que ya están aprobados para el tratamiento de otras enfermedades reumáticas como la artritis reumatoidea. Ejemplos de tales medicamentos son el rituximab (Rituxan) y abatacept (Orencia). Estos dos medicamentos no están aprobados para el tratamiento del lupus. En 2011, sin embargo, la Administración de Alimentos y Fármacos (FDA, por sus siglas en inglés) aprobó un medicamento biológico llamado belimumab (Benlysta) para el tratamiento del SLE de leve a moderado (gravedad media). Es el primer medicamento nuevo aprobado para el lupus desde 1955.

Este interesante avance en el tratamiento se produjo gracias a los estudios de investigación en pacientes, denominados [pruebas clínicas](http://www.rheumatology.org/practice/clinical/patients/diseases_and_conditions/clinicalresearchtrials.asp) . Dicho avance ofrece la esperanza de que algunos de los otros medicamentos que los investigadores están probando en pacientes ayudarán a tratar el lupus. También subraya la necesidad de que los pacientes con lupus participen en los estudios.

**Tratamiento combinado.** Los prestadores de atención de salud pueden combinar algunos medicamentos para controlar el lupus y prevenir el daño tisular.

Cada tratamiento tiene riesgos y beneficios. La mayoría de los medicamentos inmunosupresores, por ejemplo, pueden causar efectos secundarios importantes. Los efectos secundarios de estos medicamentos pueden incluir un aumento del riesgo de infecciones, así como náuseas, vómitos, pérdida de cabello, diarrea, hipertensión arterial y osteoporosis (huesos frágiles). Los reumatólogos pueden disminuir la dosis de un medicamento o dejar de recetarlo debido a efectos secundarios, o cuando la enfermedad entra en remisión. Como resultado de ello, es importante hacerse exámenes de salud y análisis de laboratorio frecuentes y exhaustivos a fin de realizar el seguimiento de sus síntomas y cambiar su tratamiento cuando sea necesario.

## Mayor impacto del lupus en la salud

El lupus, incluso cuando no está activo, puede causar futuras complicaciones. Algunos de estos problemas pueden ser fatales. Uno de estos problemas es la aterosclerosis (obstrucción de las arterias) que se puede desarrollar en mujeres más jóvenes o puede ser más grave de lo usual. Este problema aumenta el riesgo de ataques cardíacos, insuficiencia cardíaca y accidentes cerebrovasculares. Por consiguiente, resulta esencial que los pacientes con lupus reduzcan otros factores de riesgo de enfermedades cardíacas, tales como el hábito de fumar y los niveles elevados de presión arterial y colesterol. También es importante tener un estilo de vida tan activo como sea posible.

El lupus también puede ocasionar una enfermedad renal, la cual, a su vez, puede derivar en una insuficiencia renal que requiera someterse a diálisis. Usted puede ayudar a prevenir estos graves problemas buscando tratamiento ante los primeros síntomas de una enfermedad renal. Estas señales incluyen:


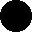
Hipertensión


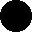

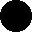
Hinchazón de pies y manos Hinchazón alrededor de los ojos


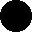
Cambios en la orina (observa sangre o espuma en la orina, va al baño más a menudo por la noche, siente dolor al orinar o tiene dificultades para hacerlo)

## Vivir con lupus

El tratamiento del lupus ha mejorado, y las personas que padecen esta enfermedad viven más tiempo. Sin embargo, sigue siendo una enfermedad crónica que puede limitar las actividades. La calidad de vida puede verse afectada a causa de síntomas como la fatiga y el dolor de las articulaciones. Además, algunas personas no responden a ciertos tratamientos. Asimismo, no puede predecir cuándo aparecerá el lupus. Estos problemas pueden derivar en depresión, ira, desesperanza o falta de voluntad para seguir luchando.

La mejor manera de controlar el lupus es seguir estos consejos.


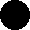
**Forme un sistema de apoyo.** Una buena relación entre médico y paciente, y el apoyo de la familia y los amigos son factores clave que pueden ayudarlo a lidiar con esta enfermedad crónica y a menudo imprevisible.


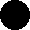
**Participe en su propio cuidado.** Tome todos los medicamentos en la forma en que se lo recetó el médico, y consúltelo con frecuencia. Aprenda tanto como pueda sobre el lupus y sus medicamentos, y qué tipo de progreso puede esperar.


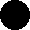
**Manténgase activo.** El ejercicio ayuda a mantener las articulaciones flexibles y puede prevenir las enfermedades cardíacas y los accidentes cerebrovasculares. Esto no implica excederse. En cambio, haga ejercicios de leves a moderados con períodos de descanso.


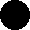
**Evite la exposición excesiva al sol.** La luz solar puede hacer que una erupción de lupus se exacerbe e incluso puede desencadenar un brote grave de la enfermedad en sí. Cuando se encuentre al aire libre en un día soleado, use ropa protectora (mangas largas, un sombrero de ala ancha) y colóquese una gran cantidad de protector solar.

**Embarazo.** Si usted es una mujer joven que padece lupus y desea tener un bebé, planifique cuidadosamente su embarazo. Con la orientación de su médico, planifique su embarazo para cuando el lupus se encuentre en un nivel de actividad bajo. Durante el embarazo, evite los medicamentos que puedan dañar a su bebé. Estos incluyen [ciclofosfamida](http://www.rheumatology.org/practice/clinical/patients/medications/cyclophosphamide.asp) , [ciclosporina](http://www.rheumatology.org/practice/clinical/patients/medications/cyclosporine.asp) y [mofetilmicofenolato](http://www.rheumatology.org/practice/clinical/patients/medications/mycophenolate.asp). Si tiene que tomar alguno de estos medicamentos o su enfermedad es muy activa, utilice métodos de control de natalidad. Para obtener más información, consulte "[Embarazo y enfermedades reumáticas](http://www.rheumatology.org/practice/clinical/patients/diseases_and_conditions/pregnancy.asp)".

**Estrógeno.** Desde hace mucho tiempo, a los reumatólogos les ha preocupado el hecho de que la hormona femenina denominada estrógeno o el tratamiento con estrógeno puede causar lupus o empeorarlo. Investigaciones recientes demostraron que los estrógenos pueden desencadenar algunas erupciones de lupus que pueden ser leves o moderadas, pero que no agravan demasiado los síntomas. Sin embargo, el estrógeno puede aumentar el riesgo de coágulos de sangre. Por lo tanto, no debe ingerir estrógeno si los exámenes de sangre muestran anticuerpos antifosfolípidos (lo que significa que ya existe un alto riesgo de formación de coágulos de sangre).

## Puntos para recordar

La mayoría de las personas con lupus pueden tener una vida normal. Para prevenir problemas graves, usted debe consultar a su reumatólogo a menudo. Esto le permite al médico realizar un seguimiento de su enfermedad y cambiar el tratamiento cuando sea necesario.

Si no dispone de un reumatólogo cerca de su domicilio, es posible que necesite que su médico de atención primaria maneje el tratamiento de lupus con la ayuda de un reumatólogo.

## El papel del reumatólogo en el tratamiento del lupus


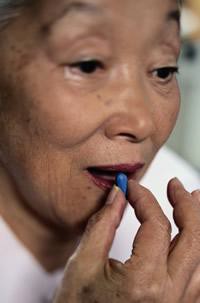


El lupus es una enfermedad compleja. Como expertos en el diagnóstico y el tratamiento de enfermedades autoinmunes como el lupus, los reumatólogos son quienes mejor pueden aconsejar a los pacientes sobre las opciones de tratamiento.

## Para buscar un reumatólogo

Para obtener una lista de reumatólogos en su área, [haga clic aquí](http://www.rheumatology.org/directory/geo.asp?aud=pat).

Obtenga más información sobre [reumatólogos](http://www.rheumatology.org/practice/clinical/patients/rheumatologist-esp.asp) y [profesionales](http://www.rheumatology.org/practice/clinical/patients/healthprofessionals/index.asp) [dedicados a la reumatología.](http://www.rheumatology.org/practice/clinical/patients/healthprofessionals/index.asp)

## Para obtener más información

El Colegio Estadounidense de Reumatología (ACR, por sus siglas en inglés) compiló esta lista con el fin de brindarle un punto de partida para que realice investigaciones adicionales por su cuenta. El ACR no respalda ni mantiene estos sitios web, ni tampoco tiene responsabilidad alguna por la información o las declaraciones allí publicadas. Lo mejor es que siempre consulte con su reumatólogo para obtener más información y antes de tomar decisiones sobre su tratamiento.

La mejor manera de controlar el lupus es involucrarse en su propio cuidado. Tome todos los medicamentos en la forma en que se lo recetó el médico, y consúltelo con frecuencia. Aprenda tanto como pueda sobre el lupus y sus medicamentos.

The Arthritis Foundation (Fundación para la Artritis) [www.arthritis.org](http://www.arthritis.org/)

The Lupus Foundation of America (Fundación Estadounidense

para el Lupus) [www.lupus.org](http://www.lupus.org/)

Lupus Research Institute (Instituto para la Investigación del Lupus) [www.LupusResearchInstitute.org](http://www.lupusresearchinstitute.org/)

National Institute of Arthritis and Musculoskeletal and Skin Diseases (Instituto Nacional de Artritis y Enfermedades Musculoesqueléticas y de la Piel) [www.niams.nih.gov/Health_Info/Lupus/default.asp](http://www.niams.nih.gov/Health_Info/Lupus/default.asp)

**Actualizado en febrero de 2013**

Escrito por la Dra. Ellen Ginzler y la Dra. Jean Tayar, y revisado por la Comisión de Marketing y Comunicaciones del Colegio Estadounidense de Reumatología.

*Esta hoja de datos para pacientes se incluye sólo con fines de educación general. Todo individuo debe consultar a un prestador de atención de salud calificado para obtener asesoramiento médico profesional, así como el diagnóstico y el tratamiento de una afección de salud.*

© 2013 American College of Rheumatology
