# Supplementary material for: Individualized decision aid for diverse women with lupus nephritis (IDEA-WON): A randomized controlled trial
Source: PLoS Med. 2019 May 8;16(5):e1002800. doi: 10.1371/journal.pmed.1002800 (PMC6505936; doi:10.1371/journal.pmed.1002800)
Supplement: S2 Text — (DOCX) [file pmed.1002800.s003.docx]

**S2 Text. Study Inclusion and Exclusion criteria**

| **Inclusion Criteria** |
| --- |
| (1) Age 18 years or older |
| (2) All racial/ethnic groups |
| (3) Females with lupus nephritis |
| (4) Currently having a flare of lupus nephritis according to expert rheumatologist and considering change or initiation of an immunosuppressive medication for lupus nephritis (current flare) or had had flare of lupus nephritis and the experience or discussion of immunosuppressive medication for lupus nephritis in the past is at the risk of a future lupus nephritis flare (future flare). |
|  |
| **Exclusion Criteria** |
| (1) Male patients with lupus nephritis |
| (2) Patients with lupus but no nephritis |
| (3) Patients having kidney flare but medication change is not considered |
| (4) Patients with end stage renal disease on dialysis |
| (5) Patients with a renal transplant or who are candidates for a renal transplant |
