# Supplementary material for: Individualized decision aid for diverse women with lupus nephritis (IDEA-WON): A randomized controlled trial
Source: PLoS Med. 2019 May 8;16(5):e1002800. doi: 10.1371/journal.pmed.1002800 (PMC6505936; doi:10.1371/journal.pmed.1002800)
Supplement: S3 Text — RCT, randomized controlled trial. (DOCX) [file pmed.1002800.s004.docx]

**S3 Text. Primary and Secondary Outcome Measures for RCT**

|  | | |
| --- | --- | --- |
| **Primary outcomes/ Time of assessment** | **Description** | **Psychometric properties** |
| Decisional Conflict Scale (DCS) [1,2] / Baseline post-intervention | 10-item patient-reported scale for lower literacy populations with 3-level response categories | Cronbach’s alpha>0.78, correlates with knowledge, regret and discontinuance, sensitive to change, predictive validity for delaying decision, meaningful change is effect size of 0.30-0.40.[2-6]  10 items with 3 response categories from yes (score 0) to unsure (score 2) to no (score 4). Score on 10-items is summed, divided by 10 and multiplied by 25 to obtain the overall score that ranges from 0 (no decisional conflict) to 100 (extreme decisional conflict).  Subscales are calculated similarly by adding the respective items: (1) uncertainty about choice, add items 9 and 10, divide by 2, multiply by 25; (2) feeling informed, add items 1, 2 and 3, divide by 3, multiply by 25; (3) Values clarity, add items 4 and 5, divide by 2, multiply by 25; and (4) feeling supported, add items 6, 7 and 8, divide by 3, multiply by 25. |
| Informed choice[7,8]/ Baseline post-intervention | Concordance between patient’s knowledge and values related to taking immunosuppressive drugs with patients’ decision to start immunosuppressive drugs | Values: test-retest coefficients >0.78, discriminates between a decision-aid and other interventions and those making different decisions.[9-11]  Knowledge: Cronbach’s alpha of 0.82-0.83, sensitive to change and discriminates between a decision-aid and usual care.[9,12,13]  Choice predisposition: test-retest coefficient >0.90, correlates with expectations, sensitive to change, discriminates between interventions.[9,12-16] |
| **Secondary outcomes** |  |  |
| Control Preferences Scale [17] / Baseline post-intervention | 2-item patient-reported assessment of whether the decision-making was collaborative or not | Correlation with patient satisfaction with care and survival and discriminates between those who feel involved in the decision versus not.[18-20] |
| Interpersonal Processes of Care [21] / Baseline post-intervention and 3-mth | 18-item patient-reported multidimensional physician-patient communication | Cronbach’s alpha 0.70, correlates with patient satisfaction and psychological state, discriminates between interventions.[22-24] |
| Audiotaped Patient-physician discussion [25] / Baseline post-intervention | Active Patient Participation Coding Scheme decoding speech acts indicating patient participation | More active patient participation is associated with more favorable health and patient-reported outcomes[26-28] and better understanding between physician and patient.[29,30] |

**References:**

1. Cohan D, Gomez E, Greenberg M, Washington S, Charlebois ED. Patient perspectives with abbreviated versus standard pre-test HIV counseling in the prenatal setting: a randomized-controlled, non-inferiority trial. PloS one. 2009;4(4):e5166. Epub 2009/04/16. doi: 10.1371/journal.pone.0005166. PubMed PMID: 19367335; PubMed Central PMCID: PMC2666158.

2. O'Connor AM. Validation of a decisional conflict scale. Medical decision making : an international journal of the Society for Medical Decision Making. 1995;15(1):25-30. PubMed PMID: 7898294.

3. Brehaut JC, O'Connor AM, Wood TJ, Hack TF, Siminoff L, Gordon E, et al. Validation of a decision regret scale. Medical decision making : an international journal of the Society for Medical Decision Making. 2003;23(4):281-92. Epub 2003/08/21. PubMed PMID: 12926578.

4. O'Connor AM, Bennett CL, Stacey D, Barry M, Col NF, Eden KB, et al. Decision aids for people facing health treatment or screening decisions. Cochrane Database Syst Rev. 2009;(3):CD001431. Epub 2009/07/10. doi: 10.1002/14651858.CD001431.pub2. PubMed PMID: 19588325.

5. Stacey D, Bennett CL, Barry MJ, Col NF, Eden KB, Holmes-Rovner M, et al. Decision aids for people facing health treatment or screening decisions. Cochrane Database Syst Rev. 2011;(10):CD001431. Epub 2011/10/07. doi: 10.1002/14651858.CD001431.pub3. PubMed PMID: 21975733.

6. Sun Q. Predicting Downstream Effects of High Decisional Conflict: Meta-analyses of the Decisional Conflict Scale. Unpublished MSc thesis. Demonstrates the predictive validity of the DCS on option knowledge, decision delay, discontinuance, and regret. 2005.

7. Marteau TM, Dormandy E, Michie S. A measure of informed choice. Health expectations : an international journal of public participation in health care and health policy. 2001;4(2):99-108. Epub 2001/05/22. PubMed PMID: 11359540.

8. Michie S, Dormandy E, Marteau TM. The multi-dimensional measure of informed choice: a validation study. Patient education and counseling. 2002;48(1):87-91. Epub 2002/09/11. PubMed PMID: 12220754.

9. O'Connor AM, Tugwell P, Wells GA, Elmslie T, Jolly E, Hollingworth G, et al. A decision aid for women considering hormone therapy after menopause: decision support framework and evaluation. Patient education and counseling. 1998;33(3):267-79. Epub 1998/09/10. PubMed PMID: 9731164.

10. O’Connor A, Tugwell P, Wells G. Testing a portable, self-administered decision aid for post menopausal women considering long-term hormone replacement therapy to prevent osteoporosis and heart disease (Abstract). Medical Decision Making. 1994;14(4):438.

11. Dodin S, Legare F, Daudelin G, Tetroe J, O'Connor A. [Making a decision about hormone replacement therapy. A randomized controlled trial]. Canadian family physician Medecin de famille canadien. 2001;47:1586-93. Epub 2001/09/20. PubMed PMID: 11561335; PubMed Central PMCID: PMC2018543.

12. Man-Son-Hing M, Laupacis A, O'Connor AM, Biggs J, Drake E, Yetisir E, et al. A patient decision aid regarding antithrombotic therapy for stroke prevention in atrial fibrillation: a randomized controlled trial. JAMA : the journal of the American Medical Association. 1999;282(8):737-43. Epub 1999/08/27. PubMed PMID: 10463708.

13. O'Connor AM, Tugwell P, Wells GA, Elmslie T, Jolly E, Hollingworth G, et al. Randomized trial of a portable, self-administered decision aid for postmenopausal women considering long-term preventive hormone therapy. Medical decision making : an international journal of the Society for Medical Decision Making. 1998;18(3):295-303. Epub 1998/07/29. PubMed PMID: 9679994.

14. O'Connor AM, Tugwell P, Wells GA, al e. A decision aid for women considering hormone therapy after menopause: Decision support framework and evaluation. Patient Education and Couselling. 1998;33:267-79.

15. Cranney A, O'Connor AM, Jacobsen MJ, Tugwell P, Adachi JD, Ooi DS, et al. Development and pilot testing of a decision aid for postmenopausal women with osteoporosis. Patient education and counseling. 2002;47(3):245-55. Epub 2002/06/29. PubMed PMID: 12088603.

16. Mitchell SL, Tetroe J, O'Connor AM. A decision aid for long-term tube feeding in cognitively impaired older persons. Journal of the American Geriatrics Society. 2001;49(3):313-6. Epub 2001/04/13. PubMed PMID: 11300244.

17. Singh JA, Sloan JA, Atherton PJ, Smith T, Hack TF, Huschka MM, et al. Preferred roles in treatment decision making among patients with cancer: a pooled analysis of studies using the Control Preferences Scale. Am J Manag Care. 2010;16(9):688-96. Epub 2010/09/30. doi: 12718 [pii]. PubMed PMID: 20873956; PubMed Central PMCID: PMCPMC3020073.

18. Degner LF, Russell CA. Preferences for treatment control among adults with cancer. Research in nursing & health. 1988;11(6):367-74. Epub 1988/12/01. PubMed PMID: 3231738.

19. Degner LF, Kristjanson LJ, Bowman D, Sloan JA, Carriere KC, O'Neil J, et al. Information needs and decisional preferences in women with breast cancer. JAMA : the journal of the American Medical Association. 1997;277(18):1485-92. Epub 1997/05/14. PubMed PMID: 9145723.

20. Adams JR, Drake RE, Wolford GL. Shared decision-making preferences of people with severe mental illness. Psychiatr Serv. 2007;58(9):1219-21. Epub 2007/09/04. doi: 58/9/1219 [pii]

10.1176/appi.ps.58.9.1219. PubMed PMID: 17766569.

21. Stewart AL, Napoles-Springer AM, Gregorich SE, Santoyo-Olsson J. Interpersonal processes of care survey: patient-reported measures for diverse groups. Health services research. 2007;42(3 Pt 1):1235-56. Epub 2007/05/11. doi: 10.1111/j.1475-6773.2006.00637.x. PubMed PMID: 17489912; PubMed Central PMCID: PMC1955252.

22. Schillinger D, Handley M, Wang F, Hammer H. Effects of self-management support on structure, process, and outcomes among vulnerable patients with diabetes: a three-arm practical clinical trial. Diabetes care. 2009;32(4):559-66. Epub 2009/01/10. doi: 10.2337/dc08-0787. PubMed PMID: 19131469; PubMed Central PMCID: PMC2660485.

23. Napoles AM, Gregorich SE, Santoyo-Olsson J, O'Brien H, Stewart AL. Interpersonal processes of care and patient satisfaction: do associations differ by race, ethnicity, and language? Health services research. 2009;44(4):1326-44. Epub 2009/06/06. doi: 10.1111/j.1475-6773.2009.00965.x. PubMed PMID: 19490162; PubMed Central PMCID: PMC2714869.

24. Schenker Y, Stewart A, Na B, Whooley MA. Depressive symptoms and perceived doctor-patient communication in the Heart and Soul study. Journal of general internal medicine. 2009;24(5):550-6. Epub 2009/03/11. doi: 10.1007/s11606-009-0937-5. PubMed PMID: 19274477; PubMed Central PMCID: PMC2669866.

25. Street RL, Jr., Millay B. Analyzing patient participation in medical encounters. Health communication. 2001;13(1):61-73. Epub 2001/05/24. doi: 10.1207/S15327027HC1301_06. PubMed PMID: 11370924.

26. Ward MM, Sundaramurthy S, Lotstein D, Bush TM, Neuwelt CM, Street RL, Jr. Participatory patient-physician communication and morbidity in patients with systemic lupus erythematosus. Arthritis and rheumatism. 2003;49(6):810-8. Epub 2003/12/16. doi: 10.1002/art.11467. PubMed PMID: 14673968.

27. Street RL, Jr., Voigt B. Patient participation in deciding breast cancer treatment and subsequent quality of life. Medical decision making : an international journal of the Society for Medical Decision Making. 1997;17(3):298-306. Epub 1997/07/01. PubMed PMID: 9219190.

28. Street RL, Jr., Piziak VK, Carpentier WS, Herzog J, Hejl J, Skinner G, et al. Provider-patient communication and metabolic control. Diabetes care. 1993;16(5):714-21. Epub 1993/05/01. PubMed PMID: 8495610.

29. Street RL, Jr., Richardson MN, Cox V, Suarez-Almazor ME. (Mis)understanding in patient-health care provider communication about total knee replacement. Arthritis and rheumatism. 2009;61(1):100-7. Epub 2009/01/01. doi: 10.1002/art.24371. PubMed PMID: 19116969.

30. Street RL, Jr., Haidet P. How well do doctors know their patients? Factors affecting physician understanding of patients' health beliefs. Journal of general internal medicine. 2011;26(1):21-7. Epub 2010/07/24. doi: 10.1007/s11606-010-1453-3. PubMed PMID: 20652759; PubMed Central PMCID: PMC3024116.
