# Supplementary material for: Individualized decision aid for diverse women with lupus nephritis (IDEA-WON): A randomized controlled trial
Source: PLoS Med. 2019 May 8;16(5):e1002800. doi: 10.1371/journal.pmed.1002800 (PMC6505936; doi:10.1371/journal.pmed.1002800)
Supplement: S4 Text — (DOCX) [file pmed.1002800.s005.docx]

**S4 Text. Detailed methods: Treatment Scenarios for Lupus Decision-Aid and Details for calculation of Informed choice**

**Treatment Scenarios for Decision-aid**

Scenario A (Treatment induction in immunosuppressive-naive): This is applicable to patients with newly diagnosed lupus nephritis or having history of lupus nephritis with deteriorating renal function, who are currently not on any immunosuppressive medication (real) or with stable renal function currently not requiring immunosuppressive drug, but at the risk of future flare requiring induction therapy (hypothetical). The decision-aid tool for this scenario will provide information about two medication choices: cyclophosphamide (cytoxan) vs. mycophenolate mofetil (cellcept).

Scenario B (Treatment maintenance in azathioprine failure): This is usually applicable to patients with lupus nephritis who are currently on azathioprine (Imuran) and have deteriorating renal function (azathioprine failure; real) or stable renal function (hypothetical). The decision-aid tool for this scenario will provide information about two medication choices: cyclophosphamide (cytoxan) vs. mycophenolate mofetil (cellcept).

Scenario C (Treatment maintenance in azathioprine and mycophenolate mofetil failure): This is usually applicable to patients with lupus nephritis who have failed (or will fail) azathioprine (Imuran) and mycophenolate mofetil (cellcept) therapy and thus have signs of deteriorating renal function (real) or stable renal function (hypothetical). The decision-aid tool for this scenario will provide information about two medication choices: cyclophosphamide (cytoxan) vs. calcineurin inhibitors (cyclosporine, tacrolimus etc.).

Scenario D (Treatment maintenance in mycophenolate mofetil failure): This is usually applicable to patients with lupus nephritis who are currently on mycophenolate mofetil (cellcept) and have deteriorating renal function (cellcept failure; real) or stable renal function (hypothetical). The decision-aid tool for this scenario will provide information about two medication choices: cyclophosphamide (cytoxan) and azathioprine (Imuran).

**Calculation of Informed choice**

**Values:** The median value score for the sample, based on the values below, was used to divide subjects into those who favored immunosuppressives and those who were against. Patients with more positive *values* for immunosuppressives indicates that they “favor immunosuppressives” vs. those with more negative values for immunosuppressives, which indicates that they are “against immunosuppressives”.

| Negative value statements for immunosuppressives | It is not a good idea to take medicines for years. |
| --- | --- |
|  | It is not worth taking medications that may interfere with my ability to care for my family or to do my work. |
|  | It is better to try natural remedies first, before taking prescription medications for my kidney disease. |
|  | It is important to avoid medicines that interfere with my immune system. |
|  | It is important to avoid strong medicines that increase the risk of infections. |
|  |  |
| Positive value statements for immunosuppressives | It is important for lupus patients to use medicines to try and rebalance their immune systems. |
|  | Taking medicine now is important to increase my chance of being healthy in the future. |
|  | It is important to reduce my chances of being on dialysis, even if it means taking medications with a chance |
|  | It is important to treat lupus kidney disease even if you have no symptoms. |
|  | It is better to make decisions based on scientific studies than what you may hear from family or friends. |

**Knowledge**: People were provided with twenty *knowledge* items (see below) and were asked to mark them true of false. Those answering ≥75% of the twenty *knowledge* items correctly (see below) were classified as adequately knowledgeable.

| 1. Treatment for lupus kidney disease works by adjusting your immune system. |
| --- |
| *2. Puffiness of the face caused by steroids is permanent.* |
| 3. Immunosuppressive medications (Cytoxan or Cellcept) allow patients to take lower doses of steroids. |
| 4. Steroids and immunosuppressive medications work better together than either medication alone. |
| *5. Immunosuppressive medications damage the vital organs in more than half of the people who take them.* |
| 6. Steroids may cause weight gain in more than half of the people who take them. |
| 7. Immunosuppressive medications can decrease lupus flares in organs other than the kidneys. |
| *8. Lupus is an infection.* |
| 9. Immunosuppressive medications eliminate the need for dialysis in almost all patients who take them. |
| *10. The risk of developing cancer after taking immunosuppressive medications is very high.* |
| 11. The long-term use of steroids can cause damage to your body. |
| *12. Cytoxan IV infusions are given once a week.* |
| 13. Steroids cause more damage to your bones (osteoporosis) than immunosuppressive medications. |
| *14. The majority of people taking immunosuppressive medications have stomach problems.* |
| *15. The majority of people taking steroids are likely to get an infection that needs to be treated with an antibiotic.* |
| 16. Lupus kidney disease can lead to permanent damage and the need for dialysis if it is not treated. |
| *17. The best way to treat lupus kidney disease is with steroids alone* |
| *18. Lupus kidney disease can usually be treated completely in less than 3 months.* |
| *19. Steroids may cause the majority of the people who take them to feel low or sad.* |
| 20. Immunosuppressive medications are available as generic medications and are covered by most insurance plans. |
| True statements: 1, 3, 4, 6, 7, 9, 11, 13, 16, 20; *Rest of the statements are False and are in italics* |
|  |

**Choice predisposition**: Choice predisposition was assessed using a 15-point ordinal scale with the following anchors to the question, “ “, 1=Willing, 8 = Uncertain, 15=Not Willing, We classified subjects’ choices as “willing to take immunosuppressives” (1-7), “no decision” (8), or “unwilling to take immunosuppressives” (9-15).

**Informed choice aggregate from subscales**

| **Table xx: Classification criteria for informed choice** | | | | |
| --- | --- | --- | --- | --- |
|  | Willing to take immunosuppressive drugs^@^ | | Not willing to take immunosuppressive drugs | |
|  | **Values^*^** | | **Values** | |
| **Knowledge^#^** | Favor immunosuppressives | Against immunosuppressives | Favor immunosuppressives | Against immunosuppressives |
| Adequate | Informed choice | - | - | Informed choice |
| Not adequate | - | - | - | - |
| **^*^**Values: categorized as favor immunosuppressives” vs. “against immunosuppressives” | | | | |
| **^#^**Knowledge: answering ≥75% of the twenty *knowledge* items correctly will be classified as adequately knowledgeable. | | | | |
| ^@^Choice predisposition: Using a 15-point scale with 1=Willing, 8 = Uncertain, 15=Not Willing, subjects’ choices were classified as “willing to take immunosuppressives” (1-7), “no decision” (8), or “unwilling to take immunosuppressives” (9-15). | | | | |

**Informed choice (sensitivity definition):** For sensitivity analyses, the definition for values was assessed as follows. Patients were classified based on whether they had higher scores on the positive values statements that were favorable towards immunosuppressive medications (e.g., “Taking medicine now is important to increase my chance of being healthy in the future”) compared to negative value statements that were not favorable towards immunosuppressives (e.g., “It is not a good idea to take medicines for years”), as net positive or net negative values for immunosuppressive medications.
