# Supplementary material for: Individualized decision aid for diverse women with lupus nephritis (IDEA-WON): A randomized controlled trial
Source: PLoS Med. 2019 May 8;16(5):e1002800. doi: 10.1371/journal.pmed.1002800 (PMC6505936; doi:10.1371/journal.pmed.1002800)
Supplement: S5 Text — An asterisk (*) indicates that one subject was excluded due to missing pre-intervention informed choice. A double asterisk (**) indicates that no subgroup differences were statistically significant at the Bonferroni-corrected p-value (p < 0.0008). Graphical literacy: low, 0–2; high, 3–4. SAHL: low, 0–14; high, >14. Numeracy: low, 0–3; high, 4–6. Trust in physicians: low, <44; high, 44–55. SAHL, Short Assessment of Health Literacy. (DOCX) [file pmed.1002800.s006.docx]

**S5 Text. Subgroup Analyses for informed choice, main analysis (using the median value, i.e., statistical approach)***

|  | **Post-intervention**  **Decision-aid**  **n (%)** | **Post-intervention**  **Pamphlet**  **n (%)** | **Odds ratio (95% Confidence Interval)** | **p-value**** |
| --- | --- | --- | --- | --- |
| Language |  |  |  |  |
| English (n = 255) | 56 (43.8%) | 42 (33.1%) | 1.8 (1, 3.3) | 0.059 |
| Spanish (n = 42) | 6 (27.3%) | 4 (20%) | 0.9 (0.1, 7.1) | 0.898 |
| Race/ethnicity |  |  |  |  |
| Non-Hispanic Black (n = 141) | 28 (40%) | 20 (28.2%) | 1.7 (0.7, 4) | 0.216 |
| Hispanic/Latino (n = 77) | 14 (35%) | 12 (32.4%) | 0.7 (0.2, 2.4) | 0.612 |
| Non-Hispanic White (n = 44) | 14 (70%) | 5 (20.8%) | 30.9 (3.3, 291) | 0.003 |
| Asian/Other (n = 33) | 6 (33.3%) | 9 (60%) | 0.5 (0, 7.9) | 0.625 |
| SES status |  |  |  |  |
| < $40,000 (n = 169) | 35 (39.3%) | 19 (23.8%) | 1.5 (0.7, 3.4) | 0.317 |
| $40,000-$80,000 (n = 44) | 8 (44.4%) | 11 (42.3%) | 1.6 (0.3, 7.4) | 0.557 |
| $80,000 or more (n = 32) | 12 (70.6%) | 8 (53.3%) | 4.3 (0.6, 30.5) | 0.148 |
| Education |  |  |  |  |
| High school or less (n = 105) | 13 (27.7%) | 14 (24.1%) | 1 (0.4, 2.8) | 0.992 |
| Greater than high school (n = 189) | 48 (48%) | 32 (36%) | 1.8 (0.9, 3.5) | 0.103 |
| Flare Type |  |  |  |  |
| Current (n = 67) | 14 (41.2%) | 9 (27.3%) | 1.4 (0.5, 4.5) | 0.537 |
| At Risk for flare (n = 230) | 48 (41.4%) | 37 (32.5%) | 1.7 (0.9, 3.2) | 0.122 |
| Graphical literacy |  |  |  |  |
| Low (n = 255) | 42 (37.2%) | 36 (32.1%) | 1.1 (0.6, 2.2) | 0.684 |
| High (n = 70) | 20 (55.6%) | 9 (26.5%) | 11.2 (2.2, 58) | 0.004 |
| Health literacy |  |  |  |  |
| Low (n = 25) | 2 (15.4%) | 0 (0%) | 1.5 (0.1, 25.1) | 0.762 |
| High (n = 269) | 60 (44.4%) | 46 (34.3%) | 1.6 (0.9, 2.9) | 0.091 |
| Numeracy |  |  |  |  |
| Low (n = 73) | 7 (22.6%) | 12 (28.6%) | 0.7 (0.2, 2.8) | 0.643 |
| High (n = 146) | 46 (56.1%) | 22 (34.4%) | 3.2 (1.4, 7.2) | 0.006 |
| Trust in physicians categories |  |  |  |  |
| Low (n = 87) | 12 (24.5%) | 9 (23.7%) | 0.9 (0.3, 2.9) | 0.873 |
| High (n = 210) | 50 (49.5%) | 37 (33.9%) | 2.1 (1.1, 4.1) | 0.023 |
| **Table Legend:** * Note: One subject was excluded due to missing pre-intervention informed choice.  ** No subgroup differences were statistically significant at the Bonferroni-corrected p-value (p < 0.0008).  Graphical literacy: Low, 0-2, High, 3-4;  SAHL, Short Assessment of Health Literacy: Low, 0-14; High, > 14;  Numeracy: Low, 0-3; High, 4-6;  Trust in physicians: Low, < 44, High, 44-55. | | | | |
