# Supplementary material for: Individualized decision aid for diverse women with lupus nephritis (IDEA-WON): A randomized controlled trial
Source: PLoS Med. 2019 May 8;16(5):e1002800. doi: 10.1371/journal.pmed.1002800 (PMC6505936; doi:10.1371/journal.pmed.1002800)
Supplement: S6 Text — An asterisk (*) indicates that one subject was excluded due to missing pre-intervention informed choice. A double asterisk (**) indicates that no subgroup differences were statistically significant at the Bonferroni-corrected p-value (p < 0.0008). Graphical literacy: low, 0–2; high, 3–4. SAHL: low, 0–14; high, >14. Numeracy: low, 0–3; high, 4–6. Trust in physicians: low, <44; high, 44–55. SAHL, Short Assessment of Health Literacy. (DOCX) [file pmed.1002800.s007.docx]

**S6 Text. Subgroup Analyses for informed choice, sensitivity analyses (using the net positive or negative value, i.e., clinical approach)***

|  | **Post-intervention**  **Decision-aid**  **n (%)** | **Post-intervention**  **Pamphlet**  **n (%)** | **Odds ratio (95% Confidence Interval)** | **p-value**** |
| --- | --- | --- | --- | --- |
| Language |  |  |  |  |
| English (n = 255) | 67 (52.3%) | 45 (35.4%) | 2.2 (1.2, 4) | 0.009 |
| Spanish (n = 42) | 9 (40.9%) | 6 (30%) | 2.3 (0.2, 21.4) | 0.479 |
| Race/ethnicity |  |  |  |  |
| Non-Hispanic Black (n = 141) | 37 (52.9%) | 23 (32.4%) | 2.4 (1, 5.3) | 0.041 |
| Hispanic/Latino (n = 77) | 20 (50%) | 12 (32.4%) | 2.2 (0.6, 7.5) | 0.215 |
| Non-Hispanic White (n = 44) | 14 (70%) | 7 (29.2%) | 6.4 (1.4, 29.5) | 0.017 |
| Asian/Other (n = 33) | 5 (27.8%) | 9 (60%) | 0.4 (0, 7.1) | 0.513 |
| SES status |  |  |  |  |
| < $40,000 (n = 169) | 47 (52.8%) | 25 (31.3%) | 2 (0.9, 4.3) | 0.082 |
| $40,000-$80,000 (n = 44) | 11 (61.1%) | 12 (46.2%) | 2.5 (0.6, 10.2) | 0.215 |
| $80,000 or more (n = 32) | 11 (64.7%) | 5 (33.3%) | 7.3 (1, 53) | 0.050 |
| Education |  |  |  |  |
| High school or less (n = 105) | 17 (36.2%) | 16 (27.6%) | 1.4 (0.5, 3.9) | 0.526 |
| Greater than high school (n = 189) | 58 (58%) | 35 (39.3%) | 2.4 (1.2, 4.7) | 0.015 |
| Flare Type |  |  |  |  |
| Current (n = 67) | 17 (50%) | 11 (33.3%) | 1.4 (0.5, 4.5) | 0.534 |
| At Risk for flare (n = 230) | 59 (50.9%) | 40 (35.1%) | 2.4 (1.2, 4.5) | 0.009 |
| Graphical literacy |  |  |  |  |
| Low (n = 255) | 55 (48.7%) | 39 (34.8%) | 1.7 (0.9, 3.3) | 0.102 |
| High (n = 70) | 21 (58.3%) | 11 (32.4%) | 5.8 (1.5, 21.8) | 0.010 |
| Health literacy |  |  |  |  |
| Low (n = 25) | 3 (23.1%) | 0 (0%) | 2.1 (0.1, 29.8) | 0.584 |
| High (n = 269) | 73 (54.1%) | 51 (38.1%) | 2.2 (1.2, 3.9) | 0.008 |
| Numeracy |  |  |  |  |
| Low (n = 73) | 9 (29%) | 12 (28.6%) | 0.8 (0.2, 2.8) | 0.771 |
| High (n = 146) | 51 (62.2%) | 24 (37.5%) | 3.4 (1.5, 7.7) | 0.004 |
| Trust in physicians categories |  |  |  |  |
| Low (n = 87) | 17 (34.7%) | 9 (23.7%) | 1.4 (0.5, 4.1) | 0.555 |
| High (n = 210) | 59 (58.4%) | 42 (38.5%) | 2.8 (1.4, 5.4) | 0.003 |
| **Table Legend:** * Note: One subject was excluded due to missing pre-intervention informed choice.  ** No subgroup differences were statistically significant at the Bonferroni-corrected p-value (p < 0.0008).  Graphical literacy: Low, 0-2, High, 3-4;  SAHL, Short Assessment of Health Literacy: Low, 0-14; High, > 14;  Numeracy: Low, 0-3; High, 4-6;  Trust in physicians: Low, < 44, High, 44-55. | | | | |
