# Supplementary figures and images for: Individualized decision aid for diverse women with lupus nephritis (IDEA-WON): A randomized controlled trial
Source: PLoS Med. 2019 May 8;16(5):e1002800. doi: 10.1371/journal.pmed.1002800 (PMC6505936; doi:10.1371/journal.pmed.1002800)

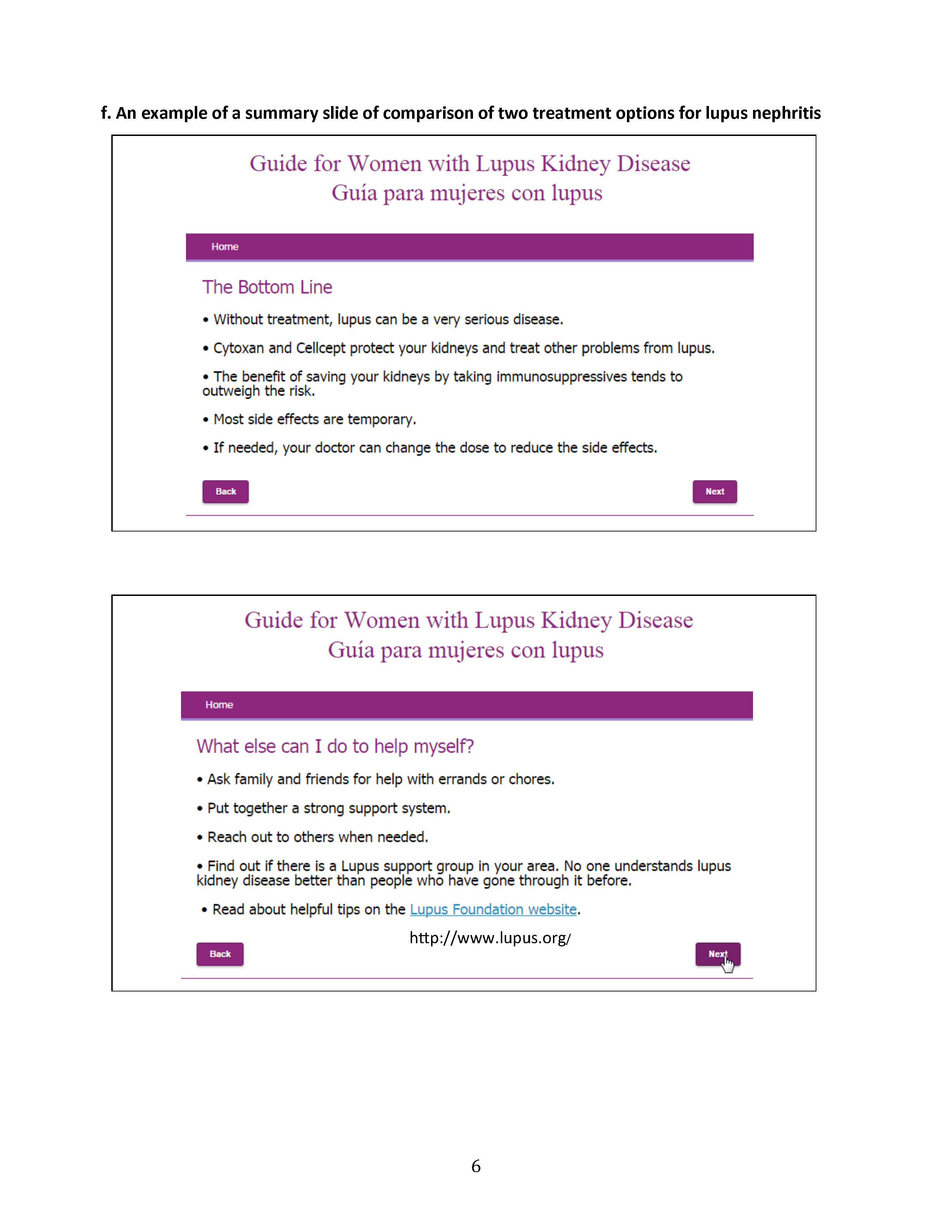

Supplement: S1 Fig — SMILE, shared decision-making in lupus electronic tool. (TIFF) [file pmed.1002800.s008.tiff]
